# The tyrosine kinase inhibitor Dasatinib reduces cardiac steatosis and fibrosis in obese, type 2 diabetic mice

**DOI:** 10.1186/s12933-023-01955-9

**Published:** 2023-08-17

**Authors:** Yue Gu, Elisa Avolio, Valeria V Alvino, Anita C Thomas, Andrew Herman, Poppy J Miller, Niall Sullivan, Ashton Faulkner, Paolo Madeddu

**Affiliations:** 1 Bristol Heart Institute, Translational Health Sciences, Bristol Medical School, University of Bristol, Upper Maudlin Street, Bristol, BS2 8HW UK; 2 School of Cellular and Molecular Medicine, University of Bristol, Upper Maudlin Street, Bristol, BS2 8HW UK; 3 University Hospitals Bristol & Weston, Bristol, UK; 4 Department of Comparative Biomedical Sciences, Royal Veterinary College, London, UK

**Keywords:** Bone marrow, Cardiac function, Dasatinib, db/db mouse model, Diabetic cardiomyopathy, Bone marrow-mesenchymal stem cells, Senescence, Senolytic agent, Steatosis, Type 2 diabetes mellitus

## Abstract

**Background:**

Cardiac steatosis is an early yet overlooked feature of diabetic cardiomyopathy. There is no available therapy to treat this condition. Tyrosine kinase inhibitors (TKIs) are used as first or second-line therapy in different types of cancer. In cancer patients with diabetes mellitus, TKIs reportedly improved glycemic control, allowing insulin discontinuation. They also reduced liver steatosis in a murine model of non-alcoholic fatty liver disease. The present study aimed to determine the therapeutic effect of the second-generation TKI Dasatinib on lipid accumulation and cardiac function in obese, type 2 diabetic mice. We also assessed if the drug impacts extra-cardiac fat tissue depots.

**Methods:**

Two studies on 21-week-old male obese leptin receptor mutant BKS.Cg-+Leprdb/+Leprdb/OlaHsd (db/db) mice compared the effect of Dasatinib (5 mg/kg) and vehicle (10% DMSO + 90% PEG-300) given via gavage once every three days for a week or once every week for four weeks. Functional and volumetric indices were studied using echocardiography. Post-mortem analyses included the assessment of fat deposits and fibrosis using histology, and senescence using immunohistochemistry and flow cytometry. The anti-adipogenic action of Dasatinib was investigated on human bone marrow (BM)-derived mesenchymal stem cells (MSCs). Unpaired parametric or non-parametric tests were used to compare two and multiple groups as appropriate.

**Results:**

Dasatinib reduced steatosis and fibrosis in the heart of diabetic mice. The drug also reduced BM adiposity but did not affect other fat depots. These structural changes were associated with improved diastolic indexes, specifically the E/A ratio and non-flow time. Moreover, Dasatinib-treated mice had lower levels of p16 in the heart compared with vehicle-treated controls, suggesting an inhibitory impact of the drug on the senescence signalling pathway. In vitro, Dasatinib inhibited human BM-MSC viability and adipogenesis commitment.

**Conclusions:**

Our findings suggest that Dasatinib opposes heart and BM adiposity and cardiac fibrosis. In the heart, this was associated with favourable functional consequences, namely improvement in an index of diastolic function. Repurposing TKI for cardiac benefit could address the unmet need of diabetic cardiac steatosis.

**Supplementary Information:**

The online version contains supplementary material available at 10.1186/s12933-023-01955-9.

## Introduction

Type 2 diabetes mellitus (T2DM) is a prevalent risk factor for cardiovascular disease [1]. Early-stage cardiac remodelling in T2DM manifests as left ventricular (LV) wall hypertrophy and reduced LV end-diastolic and end-systolic volumes. Underlying metabolic alterations comprise the loss of flexibility in myocardial substrate utilization, mitochondrial dysfunction, and the activation of inflammatory and fibrotic programs [2]. Progression of myocardial damage ultimately leads to the development of LV dysfunction and heart failure (HF), principally characterized by preserved ejection fraction [3, 4].

Cardiac steatosis, i.e., the intramyocardial accumulation of lipids, is a typical yet overlooked feature of diabetic cardiomyopathy [5]. Lipid deposits can be found in the heart of diabetic patients with normal cardiac function, suggesting that steatosis may precede and possibly contribute to the onset of diabetic cardiomyopathy [6]. Under physiologic conditions, a tight coupling between uptake and oxidation prevents the accumulation of lipids in cardiomyocytes. In T2DM, an excess free fatty acid load can overpower the cardiomyocyte’s oxidative capacity. Consequently, toxic intermediates of the non-oxidative pathway, such as ceramide, accrue causing cardiomyocyte dysfunction [7–10]. Lipotoxicity amplifies the damage from other cardiovascular risk factors, such as arterial hypertension and ischemia, through the activation of the pro-fibrotic TGF-β signalling pathway and the production of reactive oxygen species (ROS). These events eventually culminate in cardiomyocyte loss and replacement fibrosis [11]. Cardiac magnetic resonance imaging (MRI) studies have shown an association between myocardial fat content, epicardial adipose tissue volume, and a higher burden of interstitial fibrosis [12].

Whether cardiac steatosis can be prevented or reversed to halt the progression of diabetic cardiomyopathy remains under debate. Traditional antidiabetic or lipid-lowering agents, aimed at shifting the balance of cardiac metabolism from utilizing fatty acids to glucose, have shown conflicting results and may cause adverse cardiovascular events, as in the case of thiazolidinediones [13, 14]. Tyrosine kinase inhibitors (TKIs) represent a milestone in cancer therapy. Recently, TKIs have been repurposed to treat non-cancer diseases, such as autoimmune arthritis [15]. Interestingly, recent clinical evidence suggests that TKIs can improve metabolic control in cancer patients with diabetes, preserving β cell function and mass, ameliorating insulin resistance, and allowing for discontinuation of antidiabetic therapy [16, 17].

Likewise, a phase 2 clinical trial with the first-generation TKI Imatinib conducted in non-cancer patients with recent-onset type 1 diabetes demonstrated an improvement in β-cell function and peripheral insulin sensitivity [18]. Dasatinib is a second-generation TKI used to treat chronic myeloid leukemia and Philadelphia chromosome-positive acute lymphoblastic leukemia. It exhibits a more remarkable and extended kinase inhibitory potency than first-generation TKIs [19]. The association of Dasatinib and the flavonoid Quercetin attenuated adipose tissue inflammation and senescence and improved systemic metabolic function in older mice [20, 21]. In a murine model of non-alcoholic fatty liver disease, Dasatinib reportedly reduced liver steatosis, inflammation, fibrosis, and hepatocellular ballooning, by attenuating lipogenesis and inducing M2 macrophage polarization with anti-fibrotic activity [22]. Additionally, TKIs can improve the anti-lipolytic activity of insulin, thereby reducing the mobilization of free fatty acids from visceral fat depots [23]. Nonetheless, whether Dasatinib can halt or reverse cardiac steatosis remains unknown.

The present study aimed to determine the therapeutic effect of the TKI Dasatinib on myocardial lipid accumulation and cardiac function in obese, type 2 diabetic mice. We also assessed whether the drug’s action is selective to the heart or extended to traditional fat depots.

## Methods

### Protocols in mice

#### Ethics

Experimental procedures were approved by the British Home Office (PPL number: PP1377882), the University of Bristol, and were compliant with the EU Directive 2010/63/EU and principles stated in the “Guide for the Care and Use of Laboratory Animals” (Institute of Laboratory Animal Resources, 1996). Mice were housed in groups of three to six individuals in an enriched environment within a bio-secure unit under a 12 h light/dark cycle at 25℃. They were fed a standard chow diet (LabDiet) and given water ad libitum. Two studies with different follow-up durations were conducted.

#### Experimental design

In the first study (Supplementary Fig. 1), eight 21-week-old male obese leptin receptor mutant BKS.Cg-+Leprdb/+Leprdb/OlaHsd (db/db) mice (Envigo) were randomly given either Dasatinib (5 mg/kg in 10% DMSO + 90% PEG-300; Sigma Aldrich) or vehicle only (10% DMSO + 90% PEG-300) via gavage once every three days for a week. Urine glucose level was measured using One + Step® G strips. At the end of the week, the mice underwent echocardiography under isoflurane anaesthesia before being sacrificed by exsanguination. Blood was collected into EDTA (0.5 M, pH 8.0, Fisher Scientific) coated tubes. Heart, femur, and tibia were harvested. The tissue was fixed in 4% (w/v) paraformaldehyde (PFA) (Sigma-Aldrich) for histological assessment.

In the second study (Supplementary Figs. 2), 21-week-old male db/db mice were randomly allocated to receive Dasatinib (5 mg/kg) or vehicle (10% DMSO + 90% PEG-300) (n = 9 mice per group) by gavage once per week for a period of four weeks. Urine glucose level was measured as stated previously. Seven lean male C57BL/6 mice of the same age (Envigo) receiving no treatment served as a non-diabetes mellitus control reference (NDM-CTRL). At the end of the protocol, echocardiography was acquired under terminal anaesthesia, followed by blood, and tissue harvesting. Plasma was stored at -80℃ for molecular biology, and the tissues were fixed with 4% (w/v) PFA for histology. Total bone marrow (BM) cells isolated from the femur and tibia were used for flow cytometry analyses.

#### Echocardiography

Mice were studied under isoflurane anaesthesia (2.5% for induction, followed by 0.5–1.2% as appropriate to maintain a heart rate at 450 ± 25 bpm). Dimensional and functional cardiac parameters were measured using a Vevo3100 echocardiography system (Fujifilm VisualSonics Inc, Toronto, Canada).

#### Mouse bone marrow cell isolation

Both the proximal and distal ends were cut from the femur and tibia, the BM was flushed with DPBS until bone cavities were clean, and the resulting suspension was centrifuged at 200 *g* for 10 min at 25℃. After removing the supernatant, cell pellets were incubated with 1x red blood cell lysis buffer (Invitrogen) for 2 min at 4℃, washed with DPBS and centrifuged at 200 *g* for 10 min. Isolated cells were used for flow-cytometry.

#### Bones decalcification and processing for immunohistochemistry

Femoral bones were collected and cleaned from the surrounding tissues, rinsed once with PBS, and fixed with 4% (w/v) PFA for 24 h at 4℃. After fixation, tissues were decalcified with 10% (v/v) formic acid for 48 h at room temperature (RT) and embedded in OCT.

#### Immunohistochemistry of mouse heart and bone marrow

Frozen sections were post-fixed in acetone (Sigma-Aldrich) for 5 min at -20℃. After air-drying at RT for 30 min, the slides were rehydrated using distilled water for 3 min and washed with 1x PBS for 10 min. Antigen retrieval was performed in citrate buffer (pH = 6.0, microwave method, Sigma-Aldrich). After cooling, the sections were washed and non-specific binding blocked for 30 min using 20% (v/v) goat serum (Vector Laboratories) in PBS. Sections were probed with primary antibodies in 1% BSA (w/v) in PBS overnight at 4℃. After washing with PBS, sections were incubated with secondary antibody (1:200, Alexa Fluor 568-conjugated anti-mouse IgG; Invitrogen) diluted in 1x PBS for 1 h at 37℃, protected from light. The nuclei were stained with DAPI in 1x PBS for 18 min at RT, protected from light. Slides were washed with 1x PBS for 15 min, rinsed once in distilled water for 3 min, and mounted with Fluoromount-G (Invitrogen). Immunofluorescent images were acquired using a Leica SP8 LIGHTNING confocal microscope (40 x and 63 x objectives). Details of the primary and secondary antibodies used in immunohistochemistry (IHC) are listed in the Supplementary Table 1. Control stainings either omitting the primary antibody or using the isotype control are shown in Supplementary Fig. 3.

#### Lipid assessment in mouse heart and bone marrow

Imaging of neutral lipids and lipid droplets was performed on cryosections using Oil red O (ORO) (Sigma-Aldrich) staining. Images were acquired within 24 h using an Olympus BX40 light microscope (400 x and 600 x magnification).

#### Fibrosis assessment in mouse heart

Myocardial interstitial fibrosis was identified using Azan Mallory staining. Images were acquired using an Olympus BX40 light microscope (400 x magnification).

#### Multiplex assay

Mouse plasma was assayed for presence of adiponectin, interleukin 6 (IL-6), insulin, leptin, monocyte chemoattractant protein 1 (MCP-1), plasminogen activator inhibitor-1 (PAI-1), resistin, and tumor necrosis factor alpha (TNFα) using a Milliplex mouse plasma adipokine kit (Millipore) using the manufacturer’s protocol. The plates were read using a Luminex MAGPIX system (Bio-RAD, Bio-Plex® MAGPIX™ Multiplex Reader).

#### Flow cytometry analyses on mouse BM cells

Flow cytometry was employed to assess cellular senescence and the activity of mitochondria of sorted BM-MSCs. A total 500,000 cells were washed once in DPBS, spun at 200 *g* for 10 min, then stained with 100 μL Zombie Aqua dye (1:1000 in DPBS) for 20 min at RT, protected from light. After washing with cold fluorescent-activated cell sorting buffer, cells were added with TruStain FcX™ PLUS (anti-mouse CD16/32) antibody (Biolegend) for 10 min on ice with 1:200 dilution in DPBS to inhibit the non-specific binding of the immunoglobulin to the Fc receptors. Next, cells were incubated with antibodies listed in Supplementary Table 2. In addition, cells underwent MitoTracker Deep red (Invitrogen) staining with 25 nM working solution according to the manufacturer’s protocol. After staining, the cells were fixed with 2% PFA (w/v) followed by incubation in Senescence Green (Invitrogen) staining buffer for 1 h, at 37℃, without CO_2_, protected from light. Fluorescence minus one (FMO) control tubes were included in the flow cytometry panels. OneComp eBeads (eBioscience) were used to optimize the fluorescence compensation settings. The BD LSR II Fortessa X20 (Becton Dickinson, San Jose, CA, USA) and FlowJo v10.8.1 software were used for analyses.

### Protocols in human subjects

#### Ethics

The experimental procedures were covered by ethical approval from Wales Rec 4 (REC reference number: 14/WA/1005) and were compliant with the “Declaration of Helsinki” principles. Patients admitted to the Southmead Hospital (Bristol, UK) for hip reconstruction gave written informed consent to allow the use of tissue leftovers from surgery. The study was registered as an observational clinical study in the National Institute for Health Research Clinical Research Network Portfolio, UK Clinical Trials Gateway. The clinical characteristics are reported in Supplementary Table 3.

#### Isolation of BM cells

BM from the femoral head was scooped into a sterile pot and transferred into a sterile Falcon tube containing 5 mL 0.5 M EDTA, pH = 8.0, and maintained at 4℃. Samples were immediately washed 3 times with DPBS, passed through a 70 μm strainer to remove bone fragments and cell clumps, and centrifuged at 300* g* for 15 min at 25℃. Cells were resuspended in DPBS, then stratified on Ficoll Histopaque 1077 (Sigma-Aldrich) and centrifuged at 300* g* for 45 min at 25℃ (with acceleration set at 1 and without deceleration). BM mononuclear cells layered at the interphase were collected and washed with DPBS. Cell viability was assessed by trypan blue staining. A total of 1 × 10^7^ cells were seeded into flasks in αMEM basal medium (ThermoFisher Scientific) supplemented with 20% (v/v) FBS and cultured for 48 h at 37℃, 5% CO_2_. At this point, adherent cells were considered BM mesenchymal stem cells (BM-MSCs) and further expanded in αMEM + 20% (v/v) FBS.

#### Pericyte sorting

Human BM pericytes (BM-PCs) and stromal cells (BM-SCs) were obtained as previously reported [24, 25]. Once approximately 90% confluent, BM-MSCs were trypsinised and sorted for isolation of PCs. Around 1 × 10^7^ BM cells were resuspended in pre-cooled column buffer containing 0.5% (w/v) BSA and 2 mM EDTA in DPBS, labelled with CD45-conjugated microbeads (Miltenyi) for 15 min at 4℃ before subsequently being sorted by magnetic separation. The CD45-negative cells were then labelled with CD34-conjugated microbeads (Miltenyi), followed by magnet sorting. CD45^neg^ CD34^neg^ cells were labelled with CD146-conjugated microbeads (Miltenyi). The CD34^neg^ CD45^neg^ CD146^pos^ population, collected through immunomagnetic sorting, was considered BM-PCs. The remaining cells (CD45^neg^CD34^pos^ and CD45^neg^CD34^neg^CD146^neg^ populations) were pooled and considered BM-SCs. The purity of BM-PCs was assessed by immunocytochemistry staining (data not shown). Cell lines between passage 3 to passage 6 were used for subsequent experiments.

#### Oil red O staining

Cells were fixed with 4% PFA w/v for 30 min at RT and washed once with DPBS and 60% v/v isopropanol (Sigma-Aldrich), respectively. Cells were then stained with ORO working solution for 30 min at RT and washed once with 60% v/v isopropanol and distilled water. Brightfield images were taken using 5 x, 10 x and 20 x objectives of a Leica DMi1 light microscope.

#### Effect of Dasatinib treatment on adipogenic BM cells differentiation

Human BM-PCs and BM-SCs were incubated with the pro-adipogenic medium (1 μmol/L dexamethasone, 0.5 mmol/L isobutylmethylxanthine, 10 μg/mL insulin, and 1 μmol/L indomethacin in DMEM + 10% (v/v) FBS) for 21 days. During this time, with cells in the differentiation media, the protocol included an alternation of 2 days treatment with Dasatinib (1 μM) followed by 3 days without drug to allow cells to recover. Cells treated with an equal volume of DMSO served as a Vehicle group, while an untreated control group was only stimulated with adipogenic differentiation media. After the treatments, the ORO staining was performed to detect the presence of lipids. In addition, RNA was collected for qPCR analysis of relevant mRNAs, cell lysates were harvested for western blotting, and conditioned media were collected for ELISA (see below).

#### Quantitative PCR

Total RNA was extracted and purified with a miRNeasy Micro kit (QIAGEN). RNA purification was performed as per the manufacturer protocol. RNA concentration was measured using a NanoDrop 2000 Spectrophotometer (ThermoFisher Scientific). A high-capacity RNA-to-cDNA kit (ThermoFisher Scientific) was used to reverse-transcribe RNA to complementary DNA (cDNA), and synthesised cDNA was stored at -20℃. qPCR was performed using TaqMan™ Universal PCR Master Mix (Thermo Fisher Scientific) on a QuantStudio® 5 Real-Time PCR Instrument. The following adipogenesis genes were assessed: peroxisome proliferator-activated receptor gamma (*PPARG*), fatty acid binding protein 4 (*FABP4*) and adiponectin (*ADIPOQ*). Ubiquitin C (*UBC*) was used as an internal control gene. Change in expression was calculated using the 2^−ΔΔCt^ method [26]. TaqMan probes (ThermoFisher Scientific) are listed in the Supplementary Table 4.

#### Western blot analysis

Protein expression was assessed by western blot. Membranes were incubated with primary antibodies overnight at 4℃ and subsequently washed with 1x Tris-buffered saline + 0.05% Tween three times (30 min) prior to incubation with secondary antibodies for 1 h at RT (anti-rabbit or anti-mouse HRP; 1: 2000). Chemiluminescence was detected using a ChemiDoc™ MP system with Image Lab™ software (Bio-Rad). Antibodies used for western blot are listed in Supplementary Table 5.

#### ELISA

ELISA (R&D Systems) was used for detection of FABP4 and adiponectin in the conditioned medium and cell lysates, following the manufacturer protocol. The optical density was measured using a GloMax Microplate reader at 450 nm and background subtracted at 560 nm.

### Statistical analysis

Data are presented as individual values and mean ± standard error of the mean (SEM). When applicable, the D’Agostino-Pearson and Kolmogorov-Smirnov normality tests were used to check for data normal distribution. Unpaired analyses were carried out. Continuous variables normally distributed were compared using the Student’s t-test (two groups) or one-way analysis of variance (ANOVA) followed by Tukey’s multiple comparison tests (three groups). Data not normally distributed were compared using non-parametric Mann-Whitney (two groups) or Kruskal-Wallis (three groups) tests, followed by Tukey or Dunn tests as appropriate. Two-way ANOVA was used to compare the mean differences between multiple groups for in vitro experiments with human cells. A *p* value < 0.05 was considered statistically significant. All analyses used GraphPad Prism 9.4.0 (San Diego, CA, USA).

## Results

### Short-duration treatment with Dasatinib reduces myocardial lipid content without affecting cardiac function in diabetic mice

**Fig. 1:**
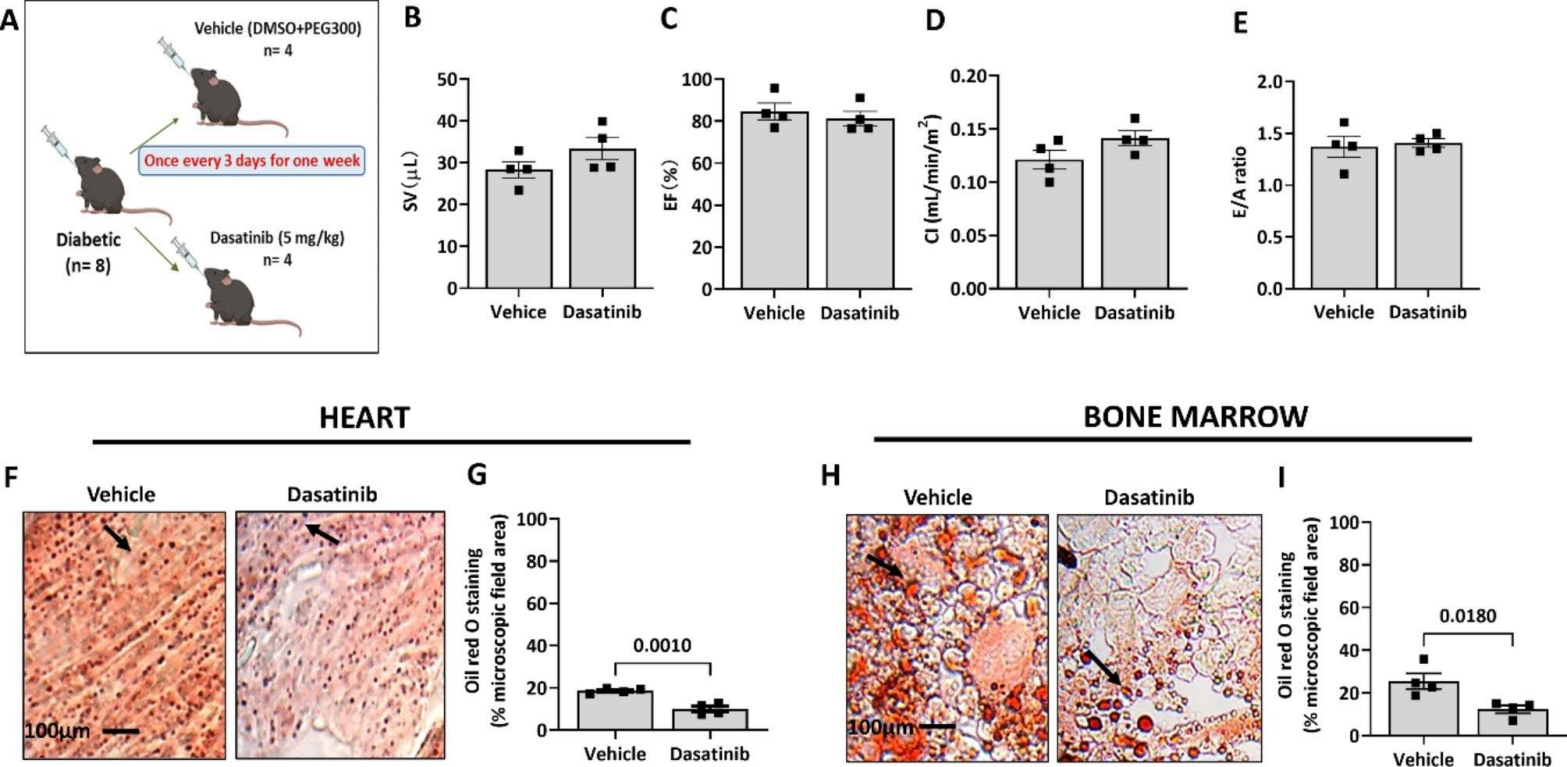
Short-duration treatment with Dasatinib reduced adiposity in the heart and bone marrow of db/db mice. (**A**) Cartoon of the experimental protocol. (**B-E**) Bar graphs showing the data of echocardiography about stroke volume (SV), ejection fraction (EF), cardiac index (CI) and E/A ratio. Illustrative images (**F**) and quantification (**G**) of ORO staining of the heart. Illustrative images (**H**) and quantification (**I**) of ORO staining of the bone marrow. Bar graphs show individual values and means ± SEM. n = 4 animals per group

In a pilot study, db/db mice were randomly assigned to receiving vehicle (10% DMSO + 90% PEG300) or Dasatinib (5 mg/kg) by gavage every 3 days (n = 4 animals/group) (Fig. 1A). There was no difference in the level of glycosuria and body weight before or after treatment (data not shown). After 1 week, echocardiography analysis documented that the two groups were similar regarding indices of systolic and diastolic function (Fig. 1B-E). Likewise, the active treatment and vehicle groups had similar plasma levels of insulin, IL-6, MCP-1, PAI-1, and resistin (data not shown). Interestingly, the IHC assessment of heart and BM adiposity using ORO staining demonstrated that db/db mice treated with Dasatinib had lower levels of lipids than the vehicle group (Fig. 1F-I). The percent of section area positive for ORO in the heart of the Dasatinib and vehicle group was 10.02 ± 1.32% and 18.65 ± 0.58%, respectively (p = 0.001). Similarly, quantifying ORO-positive cells indicated that the Dasatinib treatment reduced BM adiposity compared with vehicle (12.4 ± 1.8% vs. 25.5 ± 3.6% of microscopic field area, respectively, p = 0.018). Fluorescent microscopy imaging evidenced that Dasatinib did not impact the expression of the senescence marker p16 in the heart and BM of db/db mice (data not shown).

### Prolonged treatment with Dasatinib reduces myocardial lipid content and improves diastolic function in diabetic mice

**Fig. 2:**
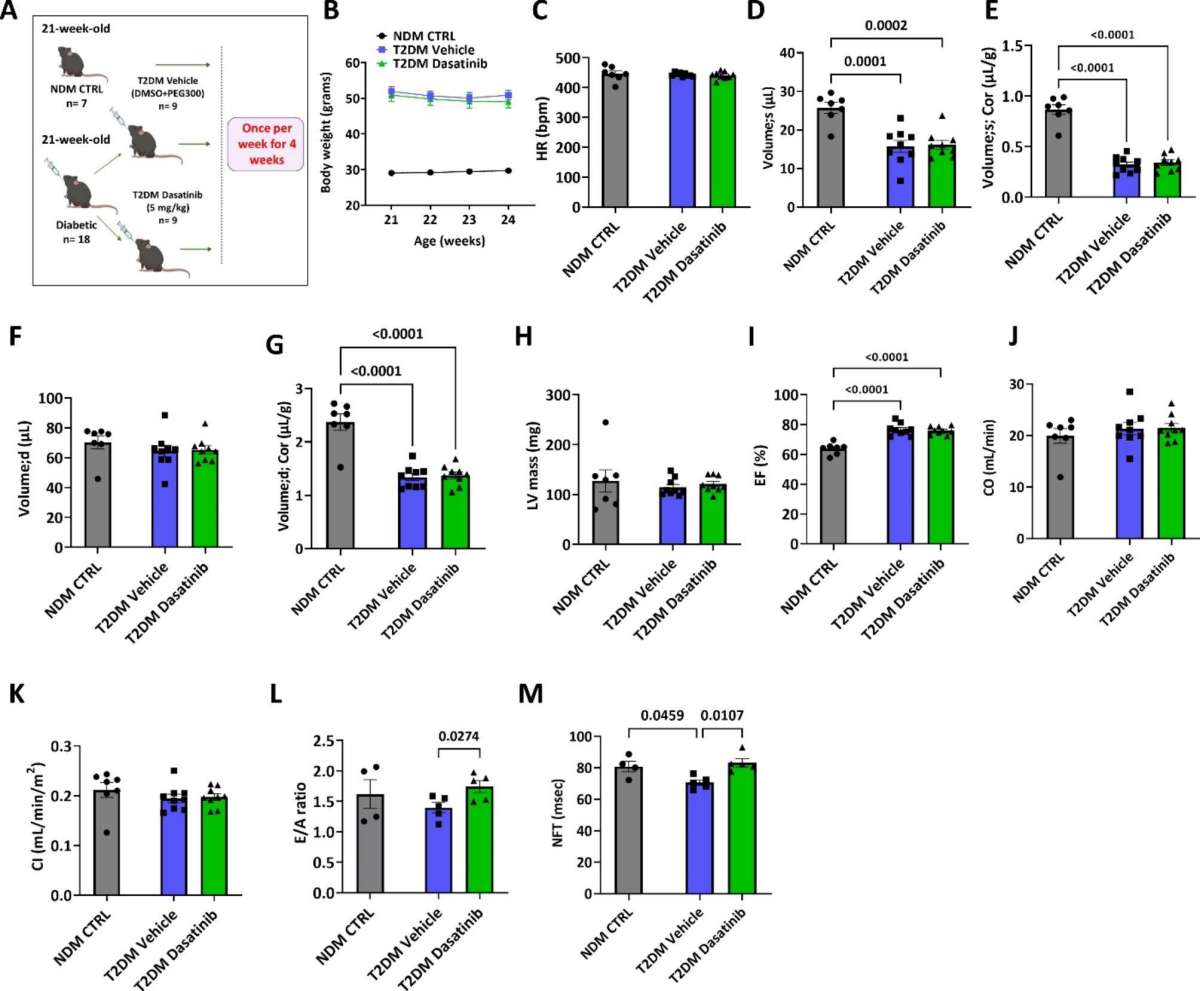
Prolonged Dasatinib treatment improves diastolic function in db/db mice. (**A**) Cartoon of the experimental protocol. (**B**) Body weight values during the follow-up. (**C**) Heart rate during echocardiography assessment. (**D-M**) Bar graphs showing the data of echocardiography regarding LV volume in systole (s) before and after correction for the LV mass (**D** & **E**), LV volume in diastole (d) before and after correction for the LV mass (**F** & **G**), LV mass (**H**), ejection fraction (**I**), cardiac output (**J**), cardiac index (**K**), E/A ratio (**L**), and NFT (**M**). Bar graphs show individual values and means ± SEM. n = 4 to 9 animals per group

Eighteen db/db mice were randomly administered with vehicle (10% DMSO + 90% PEG300) or Dasatinib (5 mg/kg) by oral gavage once per week for four weeks. A control non-diabetic group, NDM CTRL, was evaluated in parallel for reference (Fig. 2A). Measurements of urine glucose levels confirmed the stability of the diabetic status in the Dasatinib and vehicle groups. Moreover, body weight remained stable during the follow-up (Fig. 2B). Echocardiography was performed before termination to assess the cardiac function. Representative echocardiograms for animals in the three groups are shown in Supplementary Fig. 4. Heart rate was similar between groups (Fig. 2C). Compared with NDM CTRL mice, both diabetic groups showed a similar reduction in indexed LV volumes after correction for the respective ventricular mass, which is suggestive of concentric inward LV remodelling (Fig. 2D-H). Moreover, indices of contractility indicated that db/db mice had a preserved systolic function (Fig. 2I-K). Importantly, Dasatinib treatment preserved the diastolic index early (E) /late (A) mitral inflow peak velocity ratio (E/A) that was reduced in the vehicle-treated diabetic mice (p = 0.0274) (Fig. 2L). Non-flow time (NFT), another diastolic index [27], was reduced in vehicle-treated diabetic mice compared with NDM CTRL mice (p = 0.0459). Interestingly, treatment of db/db mice with Dasatinib resulted in significantly improved NFT compared with the vehicle group (p = 0.0107) (Fig. 2M). Other parameters of diastolic function were not affected by the treatment, although isovolumic relaxation time (IVRT) and aortic ejection time (AET) showed the same trend as NFT (Supplementary Fig. 5).

**Fig. 3:**
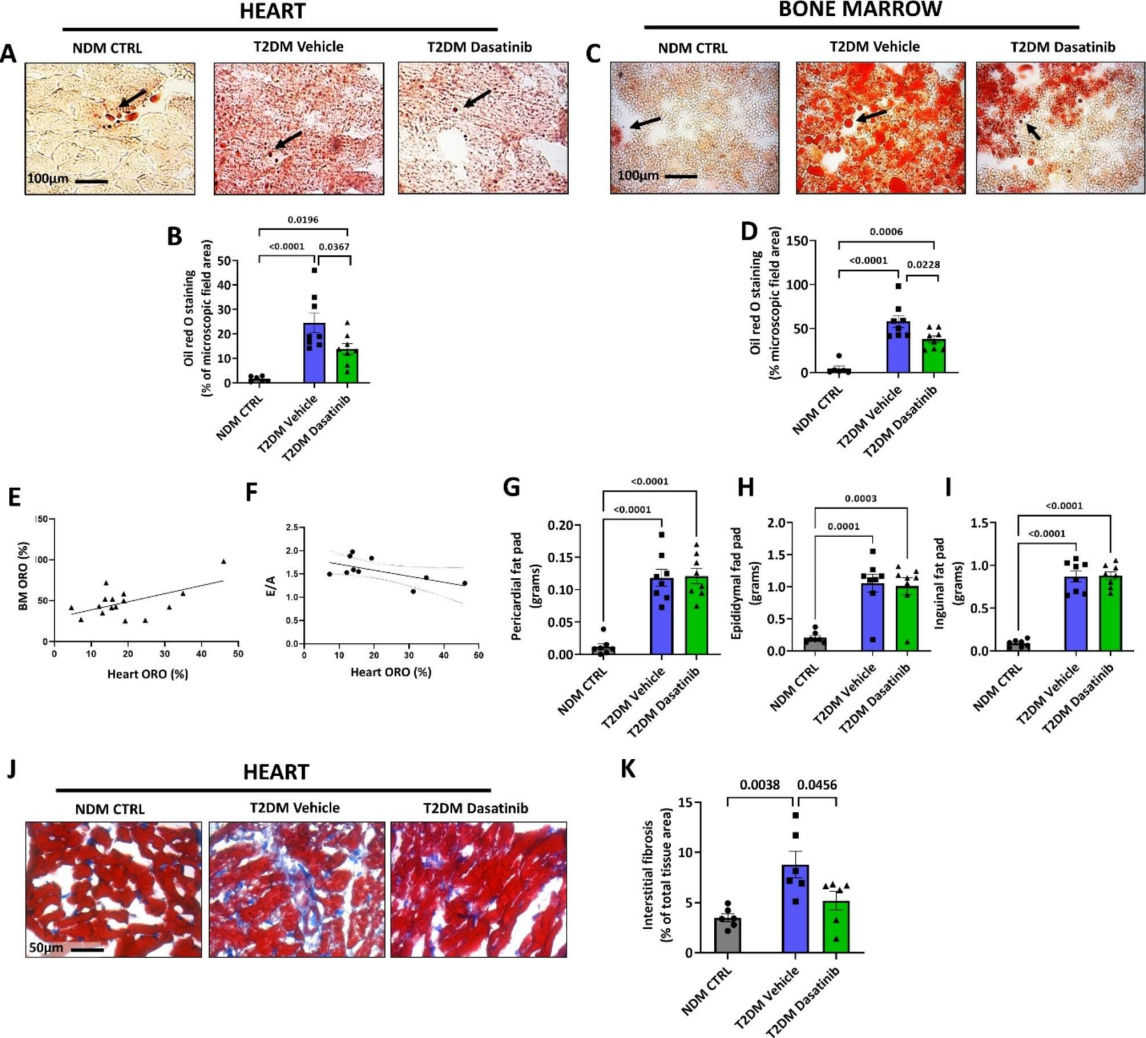
Prolonged Dasatinib treatment reduces lipid content in the heart and bone marrow, and interstitial fibrosis in the heart, of db/db mice. Representative images and bar graphs showing the effect of diabetes and Dasatinib on lipid accumulation in the heart (**A** & **B**) and bone marrow (**C** & **D**). Correlation between lipid content in the heart and bone marrow (**E**) and lipid content in the heart and E/A ratio (**F**). Effect of diabetes and Dasatinib on other fat depots (**G-I**). Representative Azan Mallory images (blue: fibrosis) and bar graph showing the effect of diabetes and Dasatinib on myocardial interstitial fibrosis (**J** & **K**). Bar graphs show individual values and means ± SEM. n = 6 to 8 animals per group

The imaging of ORO staining showed the expected lipid accumulation in the heart and BM of diabetic mice, this phenotype being blunted in mice given Dasatinib both at the heart (p = 0.0367) and BM (p = 0.0228) levels (Fig. 3A-D). There was a direct correlation between the abundance of lipids in the two districts (R^2^ = 0.32, p = 0.022) (Fig. 3E). Moreover, the amount of intramyocardial lipids was inversely correlated with the E/A ratio (R^2^ = 0.36, P = 0.05) (Fig. 3F). In contrast, Dasatinib did not impact the T2DM-induced enlargement of pericardial, epidydimal, and inguinal white fat pads (Fig. 3G-I). As expected, there was an increase in myocardial interstitial fibrosis in the hearts of diabetic mice given vehicle compared with NDM CTRL mice (p = 0.0038) (Fig. 3J**&K**). Interestingly, the percentage of tissue area occupied by fibrosis was significantly reduced in the hearts of diabetic animals receiving Dasatinib (p = 0.0456) (Fig. 3J**&K**).

Altogether, these data indicate that short-duration treatment with Dasatinib exerted protective effects against lipid accumulation in the heart and BM but did not impact white fat deposits. A prolonged treatment was necessary for an improvement in the E/A to occur. Although not necessarily indicative of a cause-effect relationship, myocardial lipid – E/A correlation suggests an association between relief of steatosis and improvement in diastolic function.

**Fig. 4:**
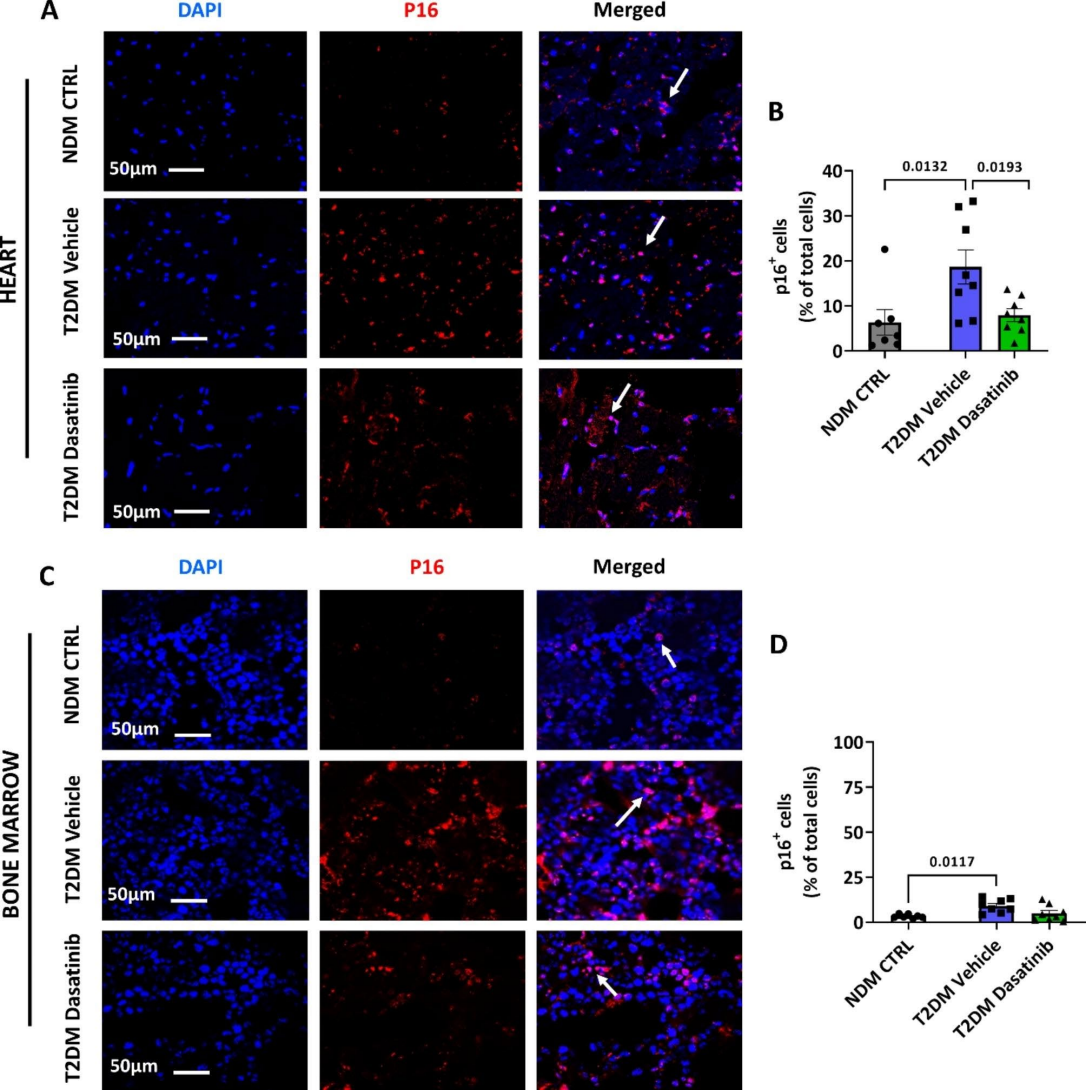
Prolonged Dasatinib treatment reduces cell senescence in the heart of db/db mice. Representative images (**A**) and bar chart (**B**) showing the levels of the senescent marker p16 in the heart, expressed as the percent of positive cells per total nuclei (stained blue by DAPI). Representative images (**C**) and bar chart (**D**) showing the level of the senescent marker p16 in the bone marrow. Bar graphs show individual values and means ± SEM. n = 7 to 8 animals per group

Next, we assessed the senescence levels in the hearts of the three groups. Quantification of p16 immunostaining indicated a lower frequency of the senescence marker in cardiac cells of Dasatinib-treated diabetic mice (7.90 ± 1.43% nuclei) compared with the vehicle-treated group (18.65 ± 3.81% nuclei) (Fig. 4A**&B**), virtually matching the low expression levels detected in cardiac cells of NDM CTRL mice (6.32 ± 2.84% nuclei). Likewise, the analysis of BM tissue confirmed an increased frequency of cells expressing p16 in vehicle-treated diabetic mice compared with NDM CTRL mice (p = 0.0117) (Fig. 4C**&D**). Unlike what was observed in the heart, in this case, the levels of cellular senescence were not corrected by Dasatinib treatment.

**Fig. 5:**
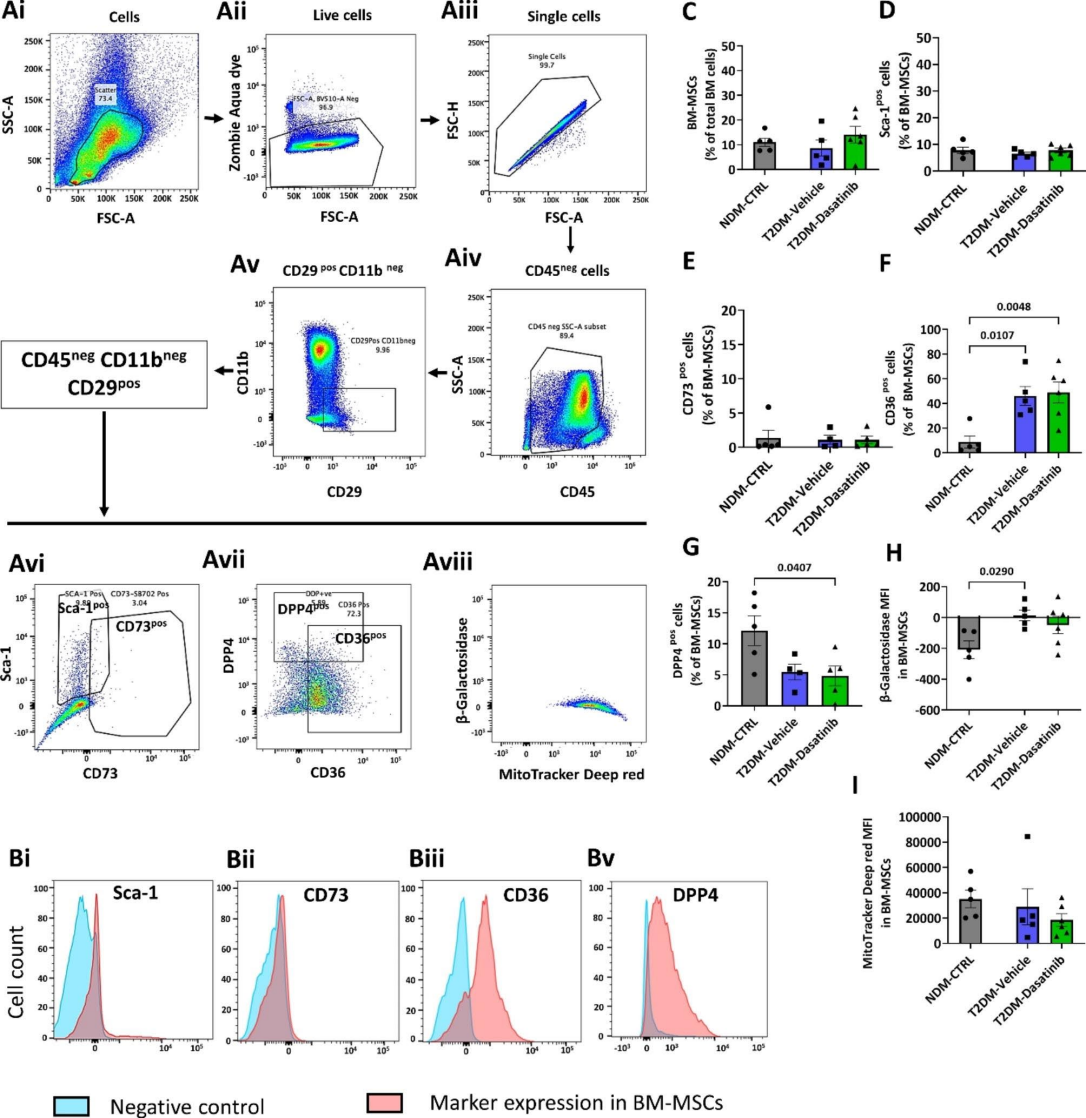
Flow cytometry analysis of mouse bone marrow mesenchymal stem cells. Freshly isolated mouse total bone marrow (BM) cells were analysed by flow cytometry for identifying (**A-E**) mesenchymal stem cells (MSC) (gating for CD45, CD11b, CD29, Sca-1 and CD73), and next for detecting senescence (gating for CD36 and β-galactosidase Senescence Green) (**F**&**H**), mobilization potential (gating for DPP4 (CD26)) (**G**) and mitochondrial activity in living cells (gating for MitoTracker Deep red) (**I**). ​The gating strategies consisted of selecting live singlet populations (Ai-Aiii), sorting for CD45^neg^CD11b^neg^CD29^pos^ MSCs (Aiv-Av), followed by examining the ratio of cell fractions positive for Sca-1, CD73, DPP4, and CD36 (Avi-Avii), and finally detecting the median fluorescence intensity (MFI) of β-galactosidase Senescence Green and MitoTracker Deep red in BM-MSCs. Representative histograms (Bi-Bv) showing markers expression in BM-MSCs (red) and their FMO controls (blue). Fluorescence intensity of β-galactosidase Senescence Green data is displayed on Bi-exponential plots. This is a hybrid scale where it is logarithmic for both positive and negative values until the 1st decade (-10 to + 10) which is displayed as linear. Bar graphs show individual values and means ± SEM. n = 4 to 6 animals per group

We next assessed if Dasatinib reduced the relative abundance and the senescence levels of BM-MSCs, the population that gives raise to adipocytes. Using flow cytometry, we verified that both CD45^neg^CD11b^neg^CD29^pos^ cells as well as the subfractions that express Sca-1 or CD73 were similarly abundant in the BM of NDM CTRL and T2DM mice and were not modified by Dasatinib (Fig. 5A-E). Looking at function-related markers, BM-MSCs from T2DM mice showed an increased frequency of the scavenger receptor CD36, which reportedly labels cells with an activated senescence-associated secretory phenotype (SASP) [28] (Fig. 5F), and a decreased frequency of DPP4, a regulator of chemokine-induced stem cell mobilization [29], in the Dasatinib group (Fig. 5G). In addition, the fluorescence signalling associated with β-galactosidase, a biomarker of replicative senescence, was upregulated in BM-MSCs of diabetic mice receiving vehicle compared with NDM CTRL mice (Fig. 5H), while there was no difference between the two diabetic groups. The MitoTracker red fluorescence intensity, which labels mitochondria of live cells, was similar between the three groups (Fig. 5I).

**Fig. 6:**
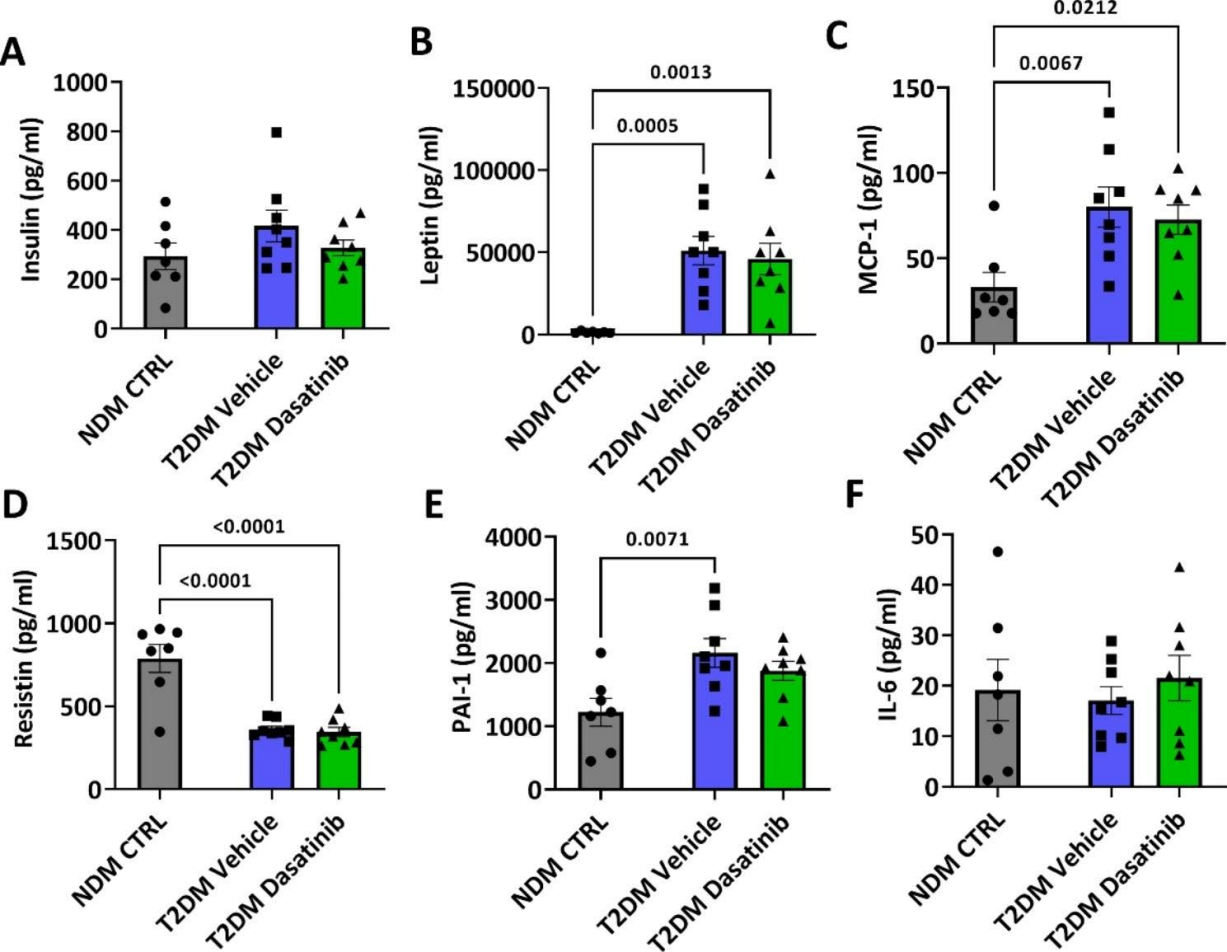
Prolonged Dasatinib treatment does not alter the plasma levels of insulin or adipokines in db/db mice. Circulating levels of Insulin (**A**), Leptin (**B**), MCP1 (**C**), Resistin (**D**), PAI-1 (**E**), and IL-6 (**F**). Bar graphs show individual values and means ± SEM. n = 7 to 8 animals per group

Finally, Dasatinib did not contrast any of the T2DM-induced changes in circulating levels of insulin, leptin, MCP-1, or resistin (Fig. 6A-D). The serine protease inhibitor, PAI-1, which is a major target of the TGF-β1/p53 senescence signalling pathway [30], was elevated in vehicle-treated diabetic mice as compared with NDM CTRL mice; in contrast, the Dasatinib-treated group did not differ from the NDM CTRL or T2DM Vehicle groups (Fig. 6E). No effect of T2DM or Dasatinib was observed regarding IL-6 (Fig. 6F). TNFα was not detected in any of the samples.

### Dasatinib blunts the adipogenic differentiation of human bone marrow mesenchymal stem cells

**Fig. 7:**
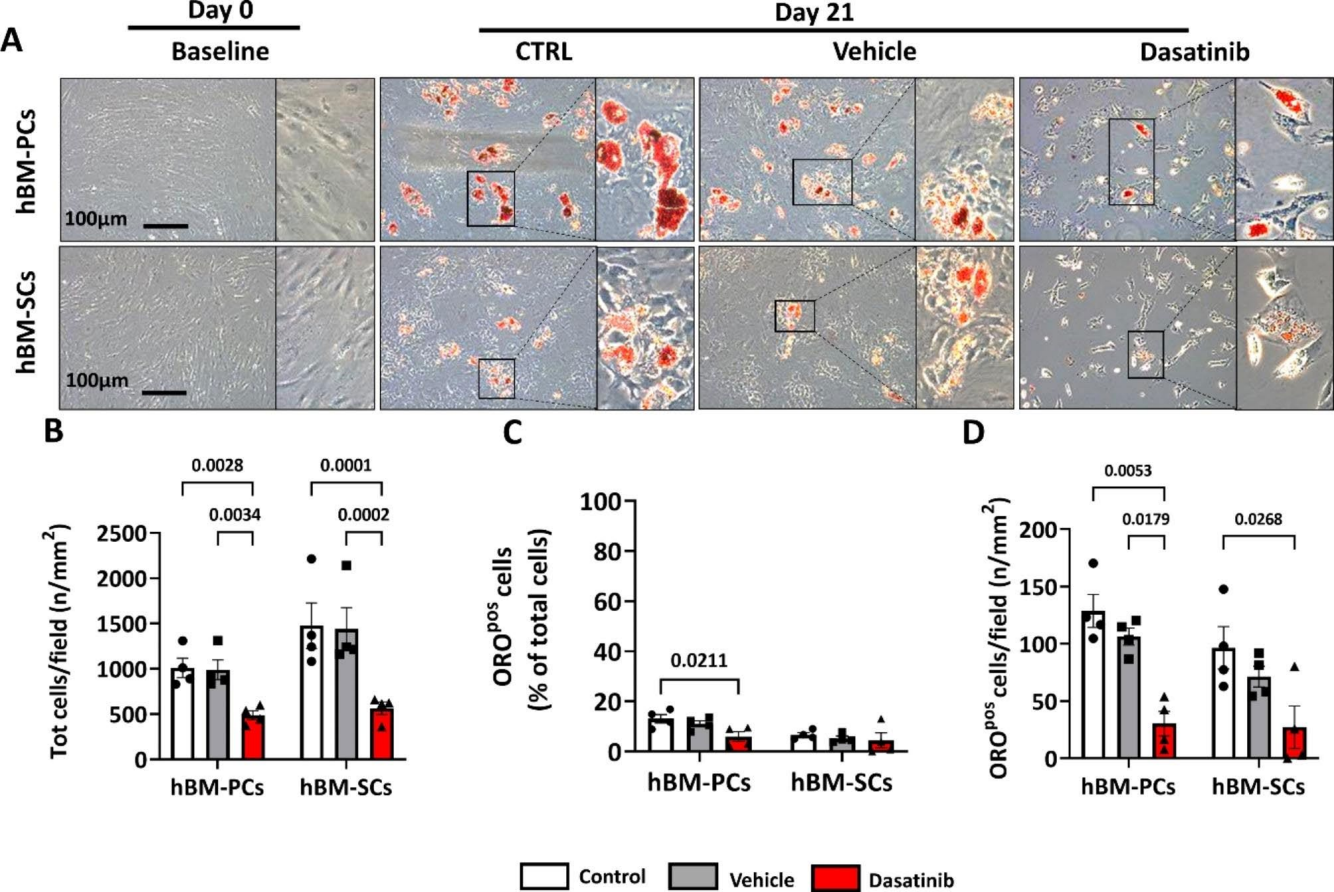
Dasatinib inhibits BM adipogenesis in vitro. (**A**) ORO staining following induction of adipogenesis in the presence of Dasatinib or vehicle. (**B**) Dasatinib reduces the total number of human bone marrow pericytes and stromal cells. (**C**-**D**) Dasatinib reduces adipogenesis either considering the prevalence over total cells or the cell density per microscopic field. Bar graphs show means ± SEM and individual values. n = 4 biological replicates per group

Next, we asked whether Dasatinib affects the adipogenic potential of BM-PCs and BM-SCs. To increase the translational impact, the study was performed on human cells, isolated from remnants of hip reconstruction. Cells were cultured in a pro-adipogenic medium, supplemented with 1 μM Dasatinib or vehicle, for 21 days (n = 4 biological replicates per group). A control group consisted of cells cultured in adipogenic medium without supplements. The ORO staining showed that Dasatinib treatment reduced the number of total cells as well as the capacity of the two cell fractions to differentiate into adipocytes (Fig. 7A). This latter data was confirmed considering either the number of ORO-positive cells per field or the fraction of ORO-positive cells per total cells (Fig. 7B-D). Therefore, Dasatinib could have both senolytic and anti-adipogenic actions.

**Fig. 8:**
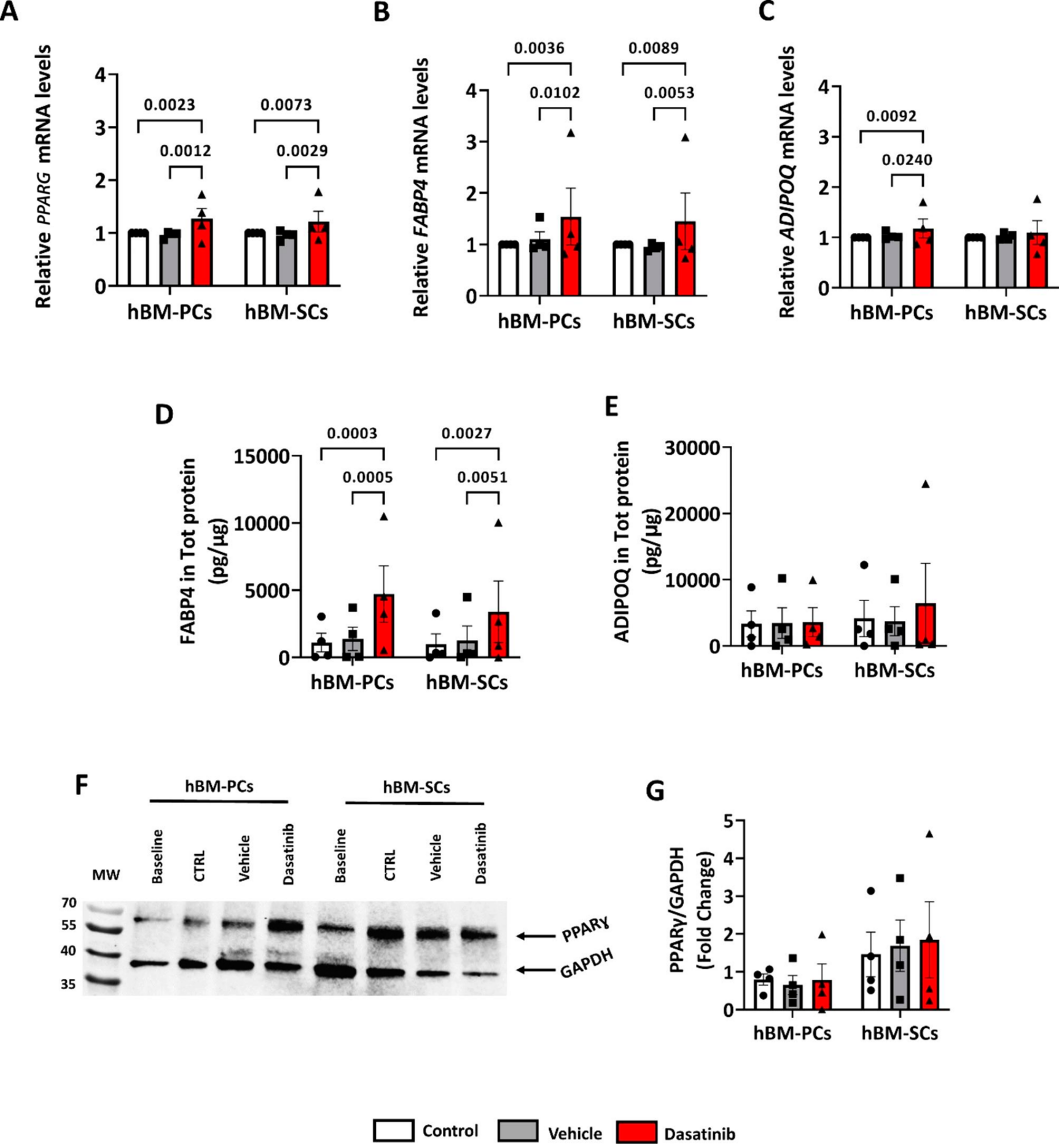
Effect of Dasatinib on the expression of adipogenesis-related gene transcripts and proteins in human cells. (**A**-**C**) Results of qPCR showing a general tendency of Dasatinib-treated cells to have increased mRNA transcripts during induced adipogenesis. (**D**-**G**) Protein level for FABP4, but not for ADIPOQ or PPARɣ, is upregulated with Dasatinib. In (G), after normalisation versus GAPDH, PPARɣ levels were expressed as a fold-change versus untreated baseline cells. Bar graphs show means ± SEM and individual values. n = 4 per group

Interestingly, the transcriptional levels of the adipogenesis genes, *PPARG* and *FABP4*, increased in Dasatinib-treated BM-MSC populations (Fig. 8A**&B)**. The qPCR results also showed that Dasatinib-treated BM-PCs expressed higher mRNA transcript levels of *ADIPOQ* (Fig. 8C**).** Next, we investigated whether the relevant proteins followed a similar trend. We measured secreted FABP4 and ADIPOQ levels in the conditioned medium after adipocyte differentiation. Western blot assessed the expression of PPARɣ in cells under different conditions, normalized by GAPDH. FABP4 was significantly increased in the conditioned medium of cells treated with Dasatinib whereas the level of ADIPOQ remained unchanged (Fig. 8D**&E)**. Furthermore, cellular expression of PPARɣ was also similar among all conditions tested (Fig. 8F**&G)**. High levels of FABP4 reportedly inhibits the expression of PPARɣ to prevent adipocyte differentiation and instead activate apoptosis [30]. These results suggest a potential PPARɣ-FABP4 feedback loop during Dasatinib treatment.

## Discussion

The present study confirms the presence of BM adiposity, cardiac steatosis and interstitial fibrosis, and LV remodelling in a murine model of T2DM. Importantly, we demonstrated for the first time that the TKI Dasatinib reduced lipid accumulation in the heart and BM of diabetic mice without affecting other adipose tissue depots or altering glycosuria. Dasatinib also attenuated cardiac fibrosis. Moreover, Dasatinib-treated mice had lower levels of p16 in the heart, suggesting an inhibitory impact of the drug on the senescence signalling pathway. In vitro studies demonstrated that Dasatinib inhibited the viability and adipogenesis commitment of human BM-MSCs.

Enlargement of white fat depots occurs during period of caloric excess and is associated with increased risk for diabetes, insulin resistance, and cardiovascular disease. Visceral fat accumulation is associated with an increased resistance against the anti-lipolytic action of insulin. Accelerated lipolysis in visceral fat leads to free fatty acid mobilisation, resulting in hyperglycaemia and hepatic insulin resistance [31]. In addition, patients with T2DM develop intraparenchymal accumulation of lipids in organs that usually contain minor amounts of fat, with harmful clinical consequences as in the case of metabolic dysfunction-associated fatty liver disease [32].

Less is known about the pathophysiological relevance of fat surrounding and infiltrating the heart. Accumulation of pericardial and epicardial fat, which is a white adipose tissue but displays also brown-fat like or beige fat features, has been associated with an increased risk for HF with preserved ejection fraction, coronary artery disease, and arrhythmias [33–35]. Lipids can also accumulate in cardiomyocytes, as initially shown in explanted hearts of patients with end-stage non-ischemic cardiomyopathy, arrhythmogenic right ventricular cardiomyopathy, and healed myocardial infarction [36]. Obesity and T2DM exacerbate this phenomenon [37]. Significantly, cardiac steatosis can precede, but also contribute to the evolution of diabetic cardiomyopathy into HF [38]. A retrospective study on T2DM patients who underwent cardiovascular computed tomography imaging demonstrated that those with more myocardial lipids had more severe LV systolic and diastolic dysfunction [39]. The circulation represents the primary source of fatty acids that become stored as triglyceride droplets within cardiomyocytes, with epicardial fat representing a secondary source. This latter assumption is based on a reported correlation between epicardial fat volume and myocardial fat content, a combination often associated with higher LV mass, interstitial fibrosis, and worse LV performance in healthy subjects and HF patients [12, 40, 41]. Therefore, reducing lipid accumulation in the heart is an urgent therapeutic target in patients with T2DM. Caloric restriction reportedly reduced myocardial lipid content in non-diabetic obese and T2DM patients [42, 43], whereas pioglitazone or metformin were ineffective [44].

Accumulating evidence suggests that TKI therapy benefits dysmetabolic disease. Imatinib, a first-generation TKI, reportedly preserved β-cell function in adult patients with recent-onset type 1 diabetes [18, 19]. An association therapy of Dasatinib, a second-generation TKI, and the flavonoid Quercetin attenuated adipose tissue inflammation and improved systemic metabolic function in older mice [20]. In addition, this drug combination had senolytic effects in white adipose tissue, as evidenced by a reduction in the expression of senescence-associated β-galactosidase, p16, and p21 [21]. Our study demonstrates that a short-duration treatment with Dasatinib was sufficient to reduce cardiac and BM steatosis. However, the treatment had to be extended to four weeks to observe a benefit on diastolic function. On the other hand, Dasatinib neither reduced classical white fat depots, thus suggesting a distinct action on the heart and BM.

Different mechanisms could be implicated in the observed cardiac benefits. Cardiomyocyte senescence is associated with disruption of the tight balance between lipid availability and oxidation [45, 46]. We have previously shown that anti-aging treatments protect the heart from T2DM-induced damage through subtle modifications in mitochondrial-related proteins and lipid metabolism [10, 47]. Dasatinib may act as a senolytic agent eliminating lipid-bearing senescent cardiomyocytes, as suggested by the reduction in p16 expression, along with an improved metabolic performance of remaining cardiomyocytes. Senolytic agents can alleviate multiple senescence-related phenotypes including activation of local and systemic SASP signalling [48]. However, in our study, Dasatinib therapy of diabetic mice did not alter the plasma levels of inflammatory adipokines [30].

A direct effect of Dasatinib on metabolic pathways is suggested by the previous finding that the TKI protected against glucose intolerance through the upregulation of Peroxisome proliferator-activated receptor gamma coactivator (PGC)-1a expression in white adipose tissue [49]. Induction of (PGC)-1a has anti-fibrotic and cardioprotective effects in T2DM [50]. Additionally, TKIs can improve the anti-lipolytic activity of insulin, thereby reducing the mobilization of free fatty acids from visceral fat depots [23]. In a murine model of non-alcoholic fatty liver disease, Dasatinib reportedly reduced liver steatosis, inflammation, fibrosis, and hepatocellular ballooning, by attenuating lipogenesis, and inducing M2 macrophage polarization with antifibrotic activity [22]. In this latter study, the TKI was given at the dosage of 4 or 8 mg/kg once daily, for four weeks [22]. We preferred to use intermittent treatment schedules to reduce potential toxic effects while preserving clinical efficacy [51].

The fat in the BM is different from the subcutaneous and visceral fat and exists in constitutive and regulated forms. Emerging evidence indicates that fat accumulation in BM may increase the risk of cardiovascular complications [52]. Dasatinib reduced BM adiposity without affecting the relative abundance of MSCs, but attenuating their senescent phenotype, as assessed by flow cytometry detection of β-galactosidase. In addition, Dasatinib impacted the commitment of cultured BM-MSCs to differentiate into adipocytes. Expressional studies showed that inhibition of adipogenesis by Dasatinib was reflected by increased RNA transcripts for *PPARG* and *ADIPOQ* genes, whereas protein expression remained unaltered. On the other hand, FABP4 was upregulated at both mRNA and secreted protein levels. Despite the lack of a typical secretory signal peptide, FABP4 is reportedly released from adipocytes in a non-classical pathway associated with lipolysis, possibly acting as an adipokine [53].

## Conclusion

Our findings suggest that Dasatinib can help combat heart steatosis and fibrosis. We can only speculate about the mechanisms underlying this benefit, which may involve Dasatinib’s metabolic and senolytic activities. Excess lipogenesis, lipolysis, and insulin resistance concur in altering the balance between energy availability and utilization with toxic consequences for the heart. By alleviating heart and liver steatosis, Dasatinib may exert synergic benefit in obese and diabetic patients. On the other hand, adverse phenomena need to be excluded during protracted exposure. In this respect, intermittent schedules may be preferable. Finally, caution is necessary regarding the diabetes model used in the present study. Furthermore, intraparenchymal fat may differ in quantity and responsiveness to treatment between male and females. A comparative study using other models, such as diabetes caused by a high-fat high-sucrose diet, and both sexes, is mandatory to confirm the findings obtained in male db/db mice.

### Electronic supplementary material

Below is the link to the electronic supplementary material.

Supplementary Material 1

## Data Availability

The data generated during the current study are available from the corresponding author on reasonable request.

## List of abbreviations

ADIPOQ: Adiponectin
AET: Aortic ejection time
ANOVA: Analysis of variance
BM: Bone marrow
DAPI: 4′,6-diamidino-2-phenylindole
db/db: BKS.Cg-+Leprdb/+Leprdb/OlaHsd
FABP4: Fatty acid binding protein 4
FMO: Fluorescence minus one
HF: Heart failure
IHC: Immunohistochemistry
IL-6: Interleukin 6
IVRT: Isovolumic relaxation time
LV: Left ventricular
MCP-1: Monocyte chemoattractant protein 1
MFI: Median fluorescence intensity
MRI: Magnetic resonance imaging
MSCs: Mesenchymal stem cells
NDM-CTRL: Non-diabetes mellitus control
NFT: Non-flow time
ORO: Oil red O
PAI-1: Plasminogen activator inhibitor-1
PBS: Phosphate-buffered saline
PCs: Pericytes
PFA: Paraformaldehyde
PGC: Peroxisome proliferator-activated receptor gamma coactivator
PPARɣ: Peroxisome proliferator-activated receptor gamma
ROS: Reactive oxygen species
SASP: Senescence-associated secretory phenotype
SCs: Stromal cells
SEM: Standard error of the mean
T2DM: Type 2 diabetes mellitus
TKIs: Tyrosine kinase inhibitors
TNFα: Tumor necrosis factor alpha
UBC: Ubiquitin C

## Declarations

Experimental procedures involving mice were approved by the British Home Office (PPL number PP1377882), the University of Bristol, and were compliant with the EU Directive 2010/63/EU and principles stated in the Guide for the Care and Use of Laboratory Animals (Institute of Laboratory Animal Resources, 1996).

## References

[1] Einarson TR, Acs A, Ludwig C, Panton UH (2018). Prevalence of cardiovascular disease in type 2 diabetes: a systematic literature review of scientific evidence from across the world in 2007–2017. Cardiovasc Diabetol.

[2] Peterson LR, Gropler RJ (2020). Metabolic and molecular imaging of the Diabetic Cardiomyopathy. Circ Res.

[3] Mordi IR. Non-invasive imaging in Diabetic Cardiomyopathy. J Cardiovasc Dev Dis 2019, 6(2). 10.3390/jcdd6020018 PMC6617232 30995812

[4] McHugh K, DeVore AD, Wu J, Matsouaka RA, Fonarow GC, Heidenreich PA, Yancy CW, Green JB, Altman N, Hernandez AF (2019). Heart failure with preserved ejection fraction and diabetes: JACC state-of-the-art review. J Am Coll Cardiol.

[5] Schulze PC, Drosatos K, Goldberg IJ (2016). Lipid use and misuse by the heart. Circ Res.

[6] Bayeva M, Sawicki KT, Ardehali H (2013). Taking diabetes to heart–deregulation of myocardial lipid metabolism in diabetic cardiomyopathy. J Am Heart Assoc.

[7] McGavock JM, Lingvay I, Zib I, Tillery T, Salas N, Unger R, Levine BD, Raskin P, Victor RG, Szczepaniak LS (2007). Cardiac steatosis in diabetes mellitus: a 1H-magnetic resonance spectroscopy study. Circulation.

[8] Park TS, Hu Y, Noh HL, Drosatos K, Okajima K, Buchanan J, Tuinei J, Homma S, Jiang XC, Abel ED (2008). Ceramide is a cardiotoxin in lipotoxic cardiomyopathy. J Lipid Res.

[9] Kovilakath A, Jamil M, Cowart LA (2020). Sphingolipids in the heart: from cradle to Grave. Front Endocrinol (Lausanne).

[10] Faulkner A, Dang Z, Avolio E, Thomas AC, Batstone T, Lloyd GR, Weber RJ, Najdekr L, Jankevics A, Dunn WB et al. Multi-Omics Analysis of Diabetic Heart Disease in the db/db model reveals potential targets for treatment by a Longevity-Associated Gene. Cells 2020, 9(5). 10.3390/cells9051283 PMC7290798 32455800

[11] Glenn DJ, Cardema MC, Ni W, Zhang Y, Yeghiazarians Y, Grapov D, Fiehn O, Gardner DG (2015). Cardiac steatosis potentiates angiotensin II effects in the heart. Am J Physiol Heart Circ Physiol.

[12] Ng ACT, Strudwick M, van der Geest RJ, Ng ACC, Gillinder L, Goo SY, Cowin G, Delgado V, Wang WYS, Bax JJ (2018). Impact of Epicardial Adipose tissue, left ventricular myocardial Fat Content, and interstitial fibrosis on myocardial contractile function. Circ Cardiovasc Imaging.

[13] Pappachan JM (2021). Efficacy and Cardiovascular Safety of Antidiabetic Medications. Curr Drug Saf.

[14] Wang L, Cai Y, Jian L, Cheung CW, Zhang L, Xia Z (2021). Impact of peroxisome proliferator-activated receptor-alpha on diabetic cardiomyopathy. Cardiovasc Diabetol.

[15] Guo K, Bu X, Yang C, Cao X, Bian H, Zhu Q, Zhu J, Zhang D (2018). Treatment Effects of the second-generation tyrosine kinase inhibitor Dasatinib on Autoimmune Arthritis. Front Immunol.

[16] Althubiti M (2022). Tyrosine kinase targeting: a potential therapeutic strategy for diabetes. Saudi J Med Med Sci.

[17] Fountas A, Diamantopoulos LN, Tsatsoulis A (2015). Tyrosine kinase inhibitors and diabetes: a Novel Treatment paradigm?. Trends Endocrinol Metab.

[18] Gitelman SE, Bundy BN, Ferrannini E, Lim N, Blanchfield JL, DiMeglio LA, Felner EI, Gaglia JL, Gottlieb PA, Long SA, et al. Imatinib therapy for patients with recent-onset type 1 diabetes: a multicentre, randomised, double-blind, placebo-controlled, phase 2 trial. Lancet Diabetes Endocrinol. 2021;9(8):502–514. 10.1016/S2213-8587(21)00139-X PMC8494464 34214479

[19] Chen R, Chen B. The role of dasatinib in the management of chronic myeloid leukemia. Drug Des Devel Ther. 2015;9:773–779. 10.2147/DDDT.S80207 PMC4330036 25709401

[20] Islam MT, Tuday E, Allen S, Kim J, Trott DW, Holland WL, Donato AJ, Lesniewski LA. Senolytic drugs, dasatinib and quercetin, attenuate adipose tissue inflammation, and ameliorate metabolic function in old age. Aging Cell. 2023;22(2):e13767. 10.1111/acel.13767 PMC9924942 36637079

[21] Murakami T, Inagaki N, Kondoh H. Cellular senescence in Diabetes mellitus: distinct senotherapeutic strategies for adipose tissue and pancreatic beta cells. Front Endocrinol (Lausanne). 2022;13:869414. 10.3389/fendo.2022.869414 PMC9009089 35432205

[22] Elsayed HRH, El-Nablaway M, Othman BH, Abdalla AM, El Nashar EM, Abd-Elmonem MM, El-Gamal R. Can dasatinib ameliorate the hepatic changes, induced by long term western diet, in mice? Ann Anat. 2021;234:151626. 10.1016/j.aanat.2020.151626 33144268

[23] Duggan BM, Foley KP, Henriksbo BD, Cavallari JF, Tamrakar AK, Schertzer JD. Tyrosine kinase inhibitors of Ripk2 attenuate bacterial cell wall-mediated lipolysis, inflammation and dysglycemia. Sci Rep. 2017;7(1):1578. 10.1038/s41598-017-01822-0 PMC5431485 28484277

[24] Mangialardi G, Ferland-McCollough D, Maselli D, Santopaolo M, Cordaro A, Spinetti G, Sambataro M, Sullivan N, Blom A, Madeddu P (2019). Bone marrow pericyte dysfunction in individuals with type 2 diabetes. Diabetologia.

[25] Ferland-McCollough D, Maselli D, Spinetti G, Sambataro M, Sullivan N, Blom A, Madeddu P (2018). MCP-1 Feedback Loop between Adipocytes and mesenchymal stromal cells causes Fat Accumulation and contributes to hematopoietic stem cell rarefaction in the bone marrow of patients with diabetes. Diabetes.

[26] Livak KJ, Schmittgen TD (2001). Analysis of relative gene expression data using real-time quantitative PCR and the 2(-Delta Delta C(T)) method. Methods.

[27] Cuijpers I, Carai P, Mendes-Ferreira P, Simmonds SJ, Mulder P, Miranda-Silva D, De Giorgio D, Pokreisz P, Heymans S, Jones EAV (2020). The effect of different anaesthetics on echocardiographic evaluation of diastolic dysfunction in a heart failure with preserved ejection fraction model. Sci Rep.

[28] Chong M, Yin T, Chen R, Xiang H, Yuan L, Ding Y, Pan CC, Tang Z, Alexander PB, Li QJ et al. CD36 initiates the secretory phenotype during the establishment of cellular senescence. EMBO Rep 2018, 19(6). 10.15252/embr.201745274 PMC5989758 29777051

[29] Fadini GP, Albiero M, Seeger F, Poncina N, Menegazzo L, Angelini A, Castellani C, Thiene G, Agostini C, Cappellari R (2013). Stem cell compartmentalization in diabetes and high cardiovascular risk reveals the role of DPP-4 in diabetic stem cell mobilopathy. Basic Res Cardiol.

[30] Samarakoon R, Higgins SP, Higgins CE, Higgins PJ. The TGF-beta1/p53/PAI-1 Signaling Axis in Vascular Senescence: role of Caveolin-1. Biomolecules 2019, 9(8). 10.3390/biom9080341 PMC6723262 31382626

[31] Carmen GY, Victor SM (2006). Signalling mechanisms regulating lipolysis. Cell Signal.

[32] Morieri ML, Vitturi N, Avogaro A, Targher G, Fadini GP, Society D-TDNotID (2021). Prevalence of hepatic steatosis in patients with type 2 diabetes and response to glucose-lowering treatments. A multicenter retrospective study in italian specialist care. J Endocrinol Invest.

[33] Iacobellis G, Bianco AC (2011). Epicardial adipose tissue: emerging physiological, pathophysiological and clinical features. Trends Endocrinol Metab.

[34] Kenchaiah S, Ding J, Carr JJ, Allison MA, Budoff MJ, Tracy RP, Burke GL, McClelland RL, Arai AE, Bluemke DA (2021). Pericardial Fat and the risk of heart failure. J Am Coll Cardiol.

[35] Milanese G, Silva M, Ledda RE, Goldoni M, Nayak S, Bruno L, Rossi E, Maffei E, Cademartiri F, Sverzellati N (2020). Validity of epicardial fat volume as biomarker of coronary artery disease in symptomatic individuals: results from the ALTER-BIO registry. Int J Cardiol.

[36] da Silva RMS, de Mello RJV (2017). Fat deposition in the left ventricle: descriptive and observacional study in autopsy. Lipids Health Dis.

[37] Dong X, Strudwick M, Wang WY, Borlaug BA, van der Geest RJ, Ng AC, Delgado V, Bax JJ, Ng AC (2023). Impact of body mass index and diabetes on myocardial fat content, interstitial fibrosis and function. Int J Cardiovasc Imaging.

[38] Iozzo P (2011). Myocardial, perivascular, and epicardial fat. Diabetes Care.

[39] Kashiwagi-Takayama R, Kozawa J, Hosokawa Y, Kato S, Kawata S, Ozawa H, Mineo R, Ishibashi C, Baden MY, Iwamoto R (2023). Myocardial fat accumulation is associated with cardiac dysfunction in patients with type 2 diabetes, especially in elderly or female patients: a retrospective observational study. Cardiovasc Diabetol.

[40] Szczepaniak LS, Dobbins RL, Metzger GJ, Sartoni-D’Ambrosia G, Arbique D, Vongpatanasin W, Unger R, Victor RG (2003). Myocardial triglycerides and systolic function in humans: in vivo evaluation by localized proton spectroscopy and cardiac imaging. Magn Reson Med.

[41] Liao PA, Lin G, Tsai SY, Wang CH, Juan YH, Lin YC, Wu MT, Yang LY, Liu MH, Chang TC (2016). Myocardial triglyceride content at 3 T cardiovascular magnetic resonance and left ventricular systolic function: a cross-sectional study in patients hospitalized with acute heart failure. J Cardiovasc Magn Reson.

[42] Hammer S, Snel M, Lamb HJ, Jazet IM, van der Meer RW, Pijl H, Meinders EA, Romijn JA, de Roos A, Smit JW (2008). Prolonged caloric restriction in obese patients with type 2 diabetes mellitus decreases myocardial triglyceride content and improves myocardial function. J Am Coll Cardiol.

[43] Viljanen AP, Karmi A, Borra R, Parkka JP, Lepomaki V, Parkkola R, Lautamaki R, Jarvisalo M, Taittonen M, Ronnemaa T (2009). Effect of caloric restriction on myocardial fatty acid uptake, left ventricular mass, and cardiac work in obese adults. Am J Cardiol.

[44] van der Meer RW, Rijzewijk LJ, de Jong HW, Lamb HJ, Lubberink M, Romijn JA, Bax JJ, de Roos A, Kamp O, Paulus WJ (2009). Pioglitazone improves cardiac function and alters myocardial substrate metabolism without affecting cardiac triglyceride accumulation and high-energy phosphate metabolism in patients with well-controlled type 2 diabetes mellitus. Circulation.

[45] Ogrodnik M, Miwa S, Tchkonia T, Tiniakos D, Wilson CL, Lahat A, Day CP, Burt A, Palmer A, Anstee QM (2017). Cellular senescence drives age-dependent hepatic steatosis. Nat Commun.

[46] Correia-Melo C, Marques FD, Anderson R, Hewitt G, Hewitt R, Cole J, Carroll BM, Miwa S, Birch J, Merz A (2016). Mitochondria are required for pro-ageing features of the senescent phenotype. EMBO J.

[47] Dang Z, Avolio E, Thomas AC, Faulkner A, Beltrami AP, Cervellin C, Carrizzo A, Maciag A, Gu Y, Ciaglia E (2020). Transfer of a human gene variant associated with exceptional longevity improves cardiac function in obese type 2 diabetic mice through induction of the SDF-1/CXCR4 signalling pathway. Eur J Heart Fail.

[48] Kirkland JL, Tchkonia T (2017). Cellular Senescence: a translational perspective. EBioMedicine.

[49] Sylow L, Long JZ, Lokurkar IA, Zeng X, Richter EA, Spiegelman BM (2016). The Cancer Drug Dasatinib increases PGC-1alpha in adipose tissue but has adverse Effects on glucose tolerance in obese mice. Endocrinology.

[50] Ihm SH, Chang K, Kim HY, Baek SH, Youn HJ, Seung KB, Kim JH (2010). Peroxisome proliferator-activated receptor-gamma activation attenuates cardiac fibrosis in type 2 diabetic rats: the effect of rosiglitazone on myocardial expression of receptor for advanced glycation end products and of connective tissue growth factor. Basic Res Cardiol.

[51] La Rosee P, Martiat P, Leitner A, Klag T, Muller MC, Erben P, Schenk T, Saussele S, Hochhaus A (2013). Improved tolerability by a modified intermittent treatment schedule of dasatinib for patients with chronic myeloid leukemia resistant or intolerant to imatinib. Ann Hematol.

[52] Santopaolo M, Gu Y, Spinetti G, Madeddu P (2020). Bone marrow fat: friend or foe in people with diabetes mellitus?. Clin Sci (Lond).

[53] Furuhashi M, Saitoh S, Shimamoto K, Miura T (2014). Fatty acid-binding protein 4 (FABP4): Pathophysiological Insights and Potent Clinical Biomarker of Metabolic and Cardiovascular Diseases. Clin Med Insights Cardiol.

