# Supplementary material for: The tyrosine kinase inhibitor Dasatinib reduces cardiac steatosis and fibrosis in obese, type 2 diabetic mice
Source: Cardiovasc Diabetol. 2023 Aug 17;22:214. doi: 10.1186/s12933-023-01955-9 (PMC10436421; doi:10.1186/s12933-023-01955-9)
Supplement: Supplementary file 1 — Supplementary Material 1 [file 12933_2023_1955_MOESM1_ESM.docx]

**The tyrosine kinase inhibitor Dasatinib reduces cardiac steatosis and fibrosis in obese, type 2 diabetic mice**

**SUPPLEMENTARY MATERIAL**

**Supplementary Figure 1: Protocol of the short-duration study in diabetic mice.**


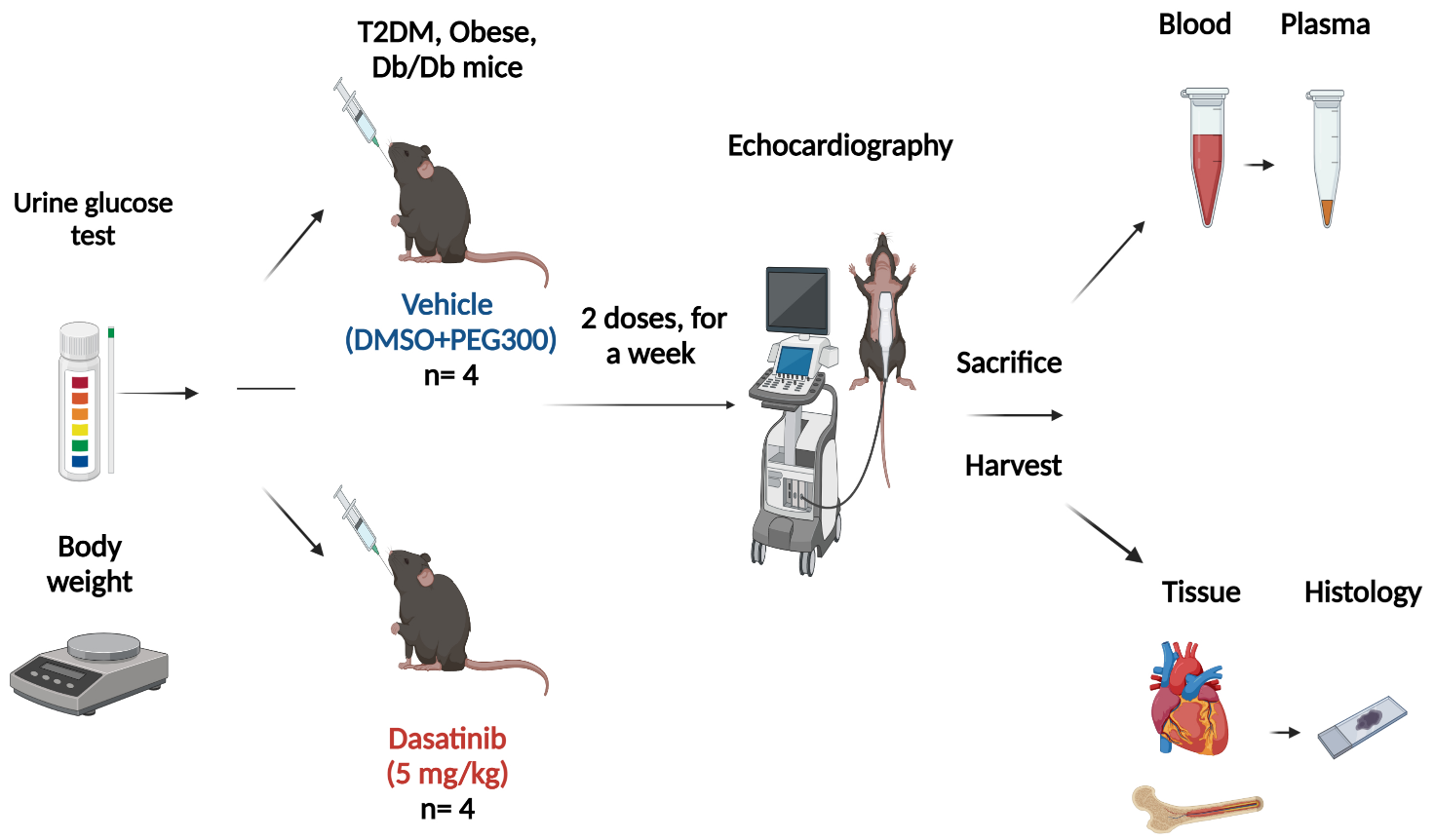


**Supplementary Figure 2: Protocol of the long-duration study in diabetic mice.**


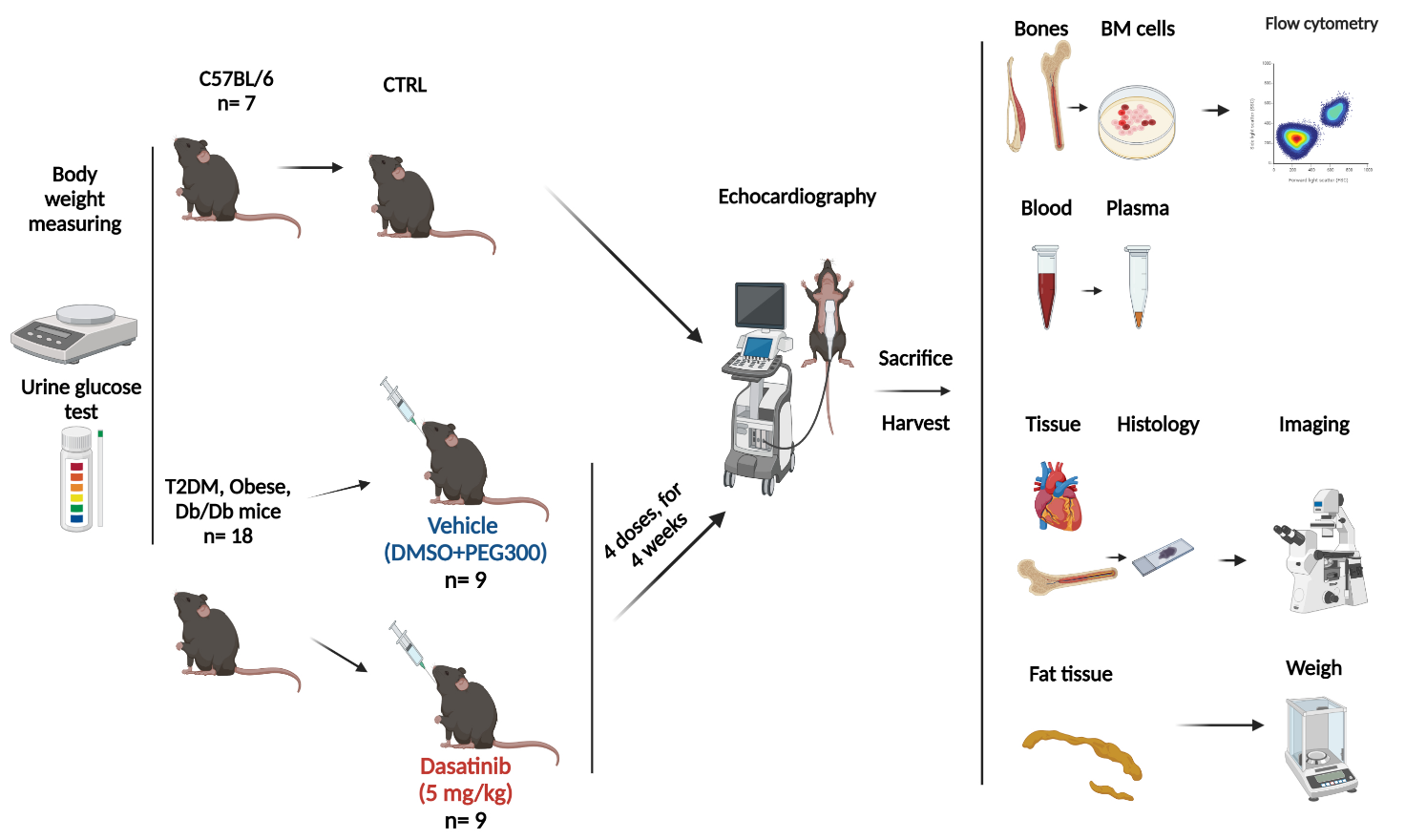


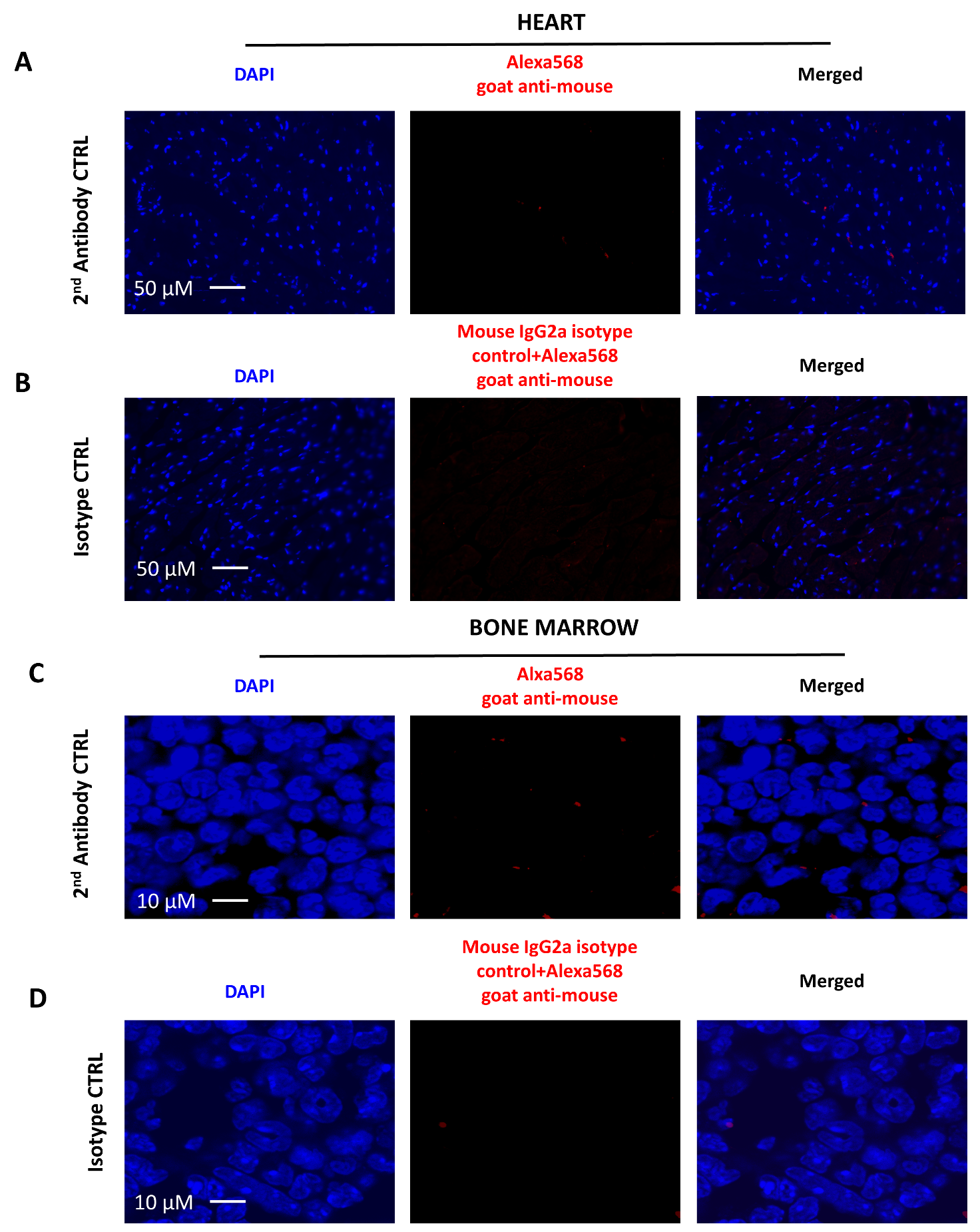


**Supplementary Figure 3: Control staining for mouse heart and bone marrow p16 immunohistochemistry study.** Representative images of control stainings either omitting the primary antibody (**A&C**) or using the mouse isotype control (**B&D**).


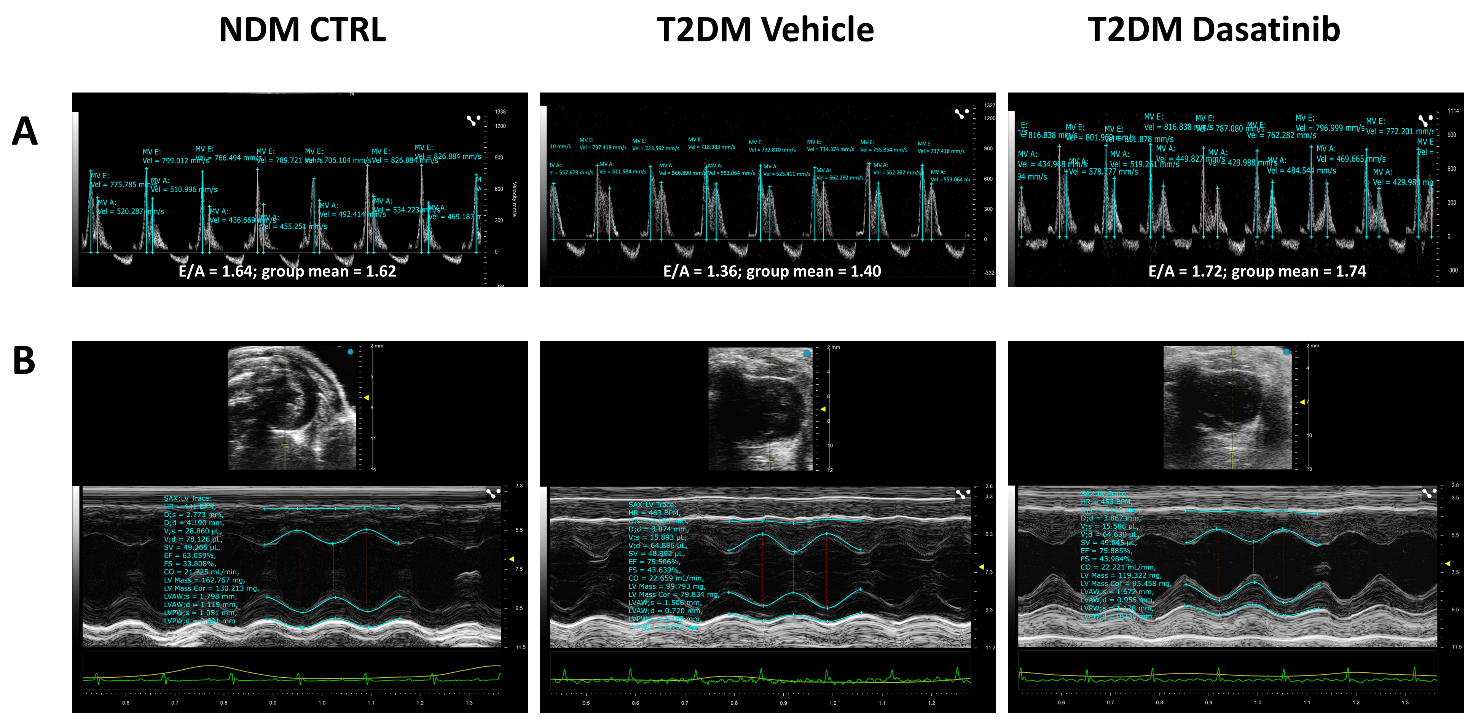


**Supplementary Figure 4: Representative echocardiograms from animals in the three experimental groups.** Images of E and A waves measurement within pulsed-wave Doppler **(A)** and M-mode traces **(B)**.


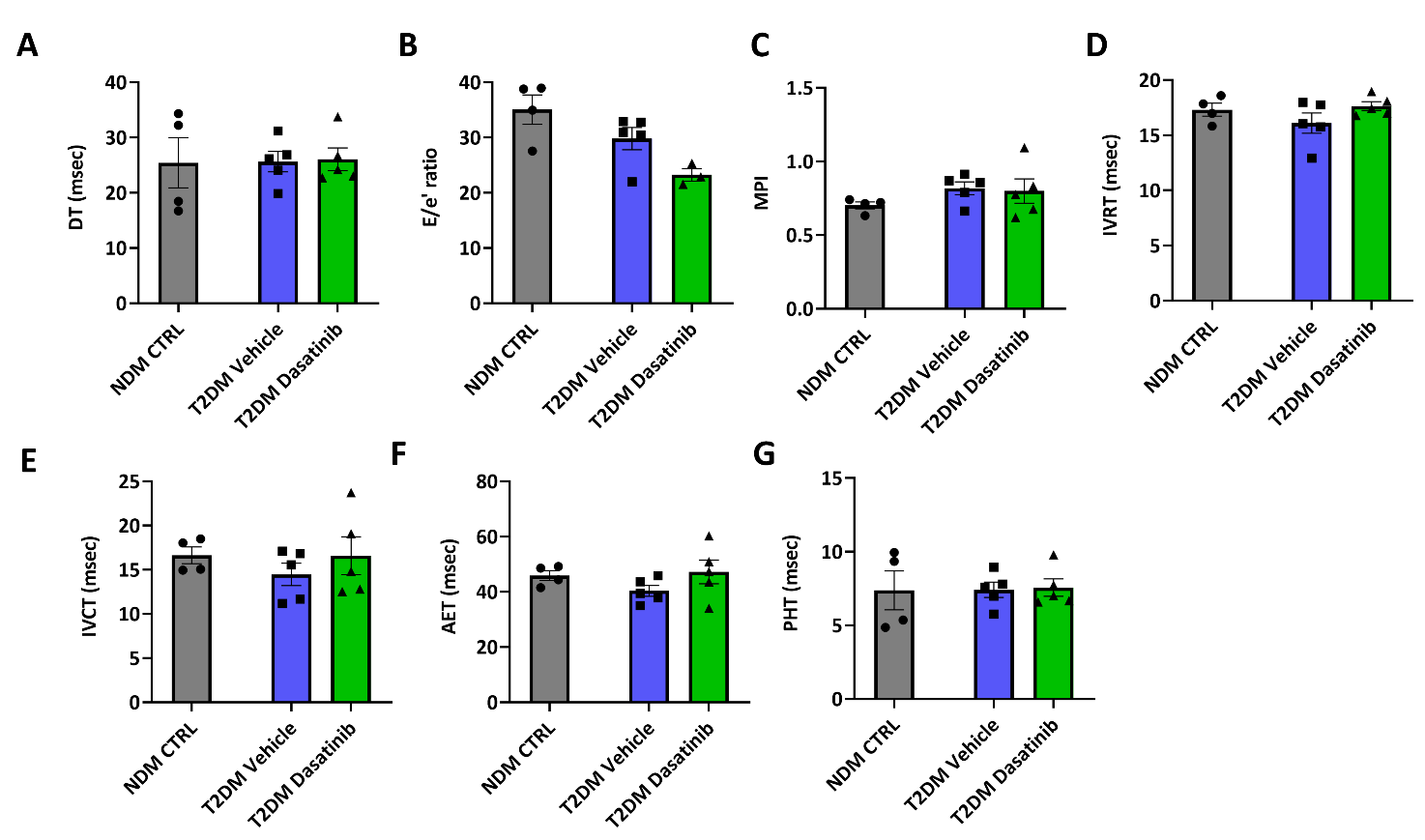


**Supplementary Figure 5: Echocardiography diastolic function parameters of prolonged Dasatinib treatment of db/db mice.** Bar graphs showing the results of mice diastolic function parameters: **(A)** DT (msec); **(B)** E/e’; **(C)** MPI; **(D)** IVRT (msec); **(E)** IVCT (msec); **(F)** AET (msec) and **(G)** PHT (msec). Abbreviations: DT = deceleration time, E/e’ ratio = early mitral inflow peak velocity/ early diastolic mitral annulus peak velocity ratio, MPI = myocardial performance index, IVRT = isovolumic relaxation time, IVCT = isovolumic contraction time, AET = aortic ejection time, PHT = mitral valve pressure half time.

Supplementary Table 1. Primary and secondary antibodies used in immunohistochemistry studies.

| **Antigen** | **Host** | **Reactivity** | **Dilution**  **(Working concentration)** | **Supplier & CAT#** | **2^nd^ Abs** |
| --- | --- | --- | --- | --- | --- |
| P16 | Mouse | Mouse | 1:50 (4 µg/mL) | Santa Cruz,  SC-1661 | Invitrogen, Alexa568 goat anti-mouse |
| Mouse IgG2a isotype control | Mouse / IgG2a | Mouse | 1:250 (4 µg/mL) | Invitrogen,  02-6200 | Invitrogen, Alexa568 goat anti-mouse |

Supplementary Table 2: List of antibodies used in flow cytometry analyses on mouse BM cells.

| **Antibody** | **Reactivity** | **Dilution** | **Fluorophores** | **Supplier** |
| --- | --- | --- | --- | --- |
| CD36 | Mouse | 1:30 | Super Bright 600 | eBioscience |
| DPP-4 | Mouse | 1:20 | PE | Biolegend |
| CD29 | Mouse | 1:10 | APC-Vio 770 | Miltenyi Biotec |
| CD45 | Mouse | 1:160 | Brilliant Violet 785 | Biolegend |
| CD73 | Mouse | 1:80 | Super Bright 702 | eBioscience |
| Sca-1 | Mouse | 1:40 | Brilliant Violet 421 | Biolegend |
| CD11b | Mouse | 1:100 | APC-Vio® 770, REAfinity™ | Miltenyi Biotec |

**Supplementary Table 3: Clinical characteristics of patients recruited to the study.**

| Sample NO. | Gender | Age | Diabetic | ICC Phenotype | Dasatinib+ Differentiation |
| --- | --- | --- | --- | --- | --- |
| BM1 | Male | 63 | No | √ | √ |
| BM2 | Male | 48 | No | √ | √ |
| BM3 | Female | 67 | No | √ | √ |
| BM4 | Male | 72 | No | √ | √ |

**Supplementary table 4: TaqMan probes used in qPCR assays.**

| **Gene** | **Species** | **Assay ID** |
| --- | --- | --- |
| *PPARɣ* | Human | Hs01115513_m1 |
| *FABP4* | Human | Hs01086177_m1 |
| *ADIPOQ* | Human | Hs00977214_m1 |
| *UBC* | Human | Hs00824723_m1 |

Supplementary Table 5: List of antibodies used for Western blot analyses.

| **Antibody** | **Reactivity** | **MW (kDa)** | **Source** | **Dilution** | **CAT#** | **Supplier** |
| --- | --- | --- | --- | --- | --- | --- |
| PPARɣ | Human | 53, 57 | Rabbit | 1:1000 | 2435 | Cell Signalling |
| GAPDH | Human | 37 | Mouse | 1:1000 | 97166 | Cell Signalling |
